# Mitochondrial DNA haplogroups and trajectories of cardiometabolic risk factors during childhood and adolescence: A prospective cohort study

**DOI:** 10.1371/journal.pone.0284226

**Published:** 2023-04-12

**Authors:** Kate N. O’Neill, Emily Aubrey, Laura D. Howe, Evie Stergiakouli, Santiago Rodriguez, Patricia M. Kearney, Linda M. O’Keeffe

**Affiliations:** 1 School of Public Health, University College Cork, Cork, Ireland; 2 MRC Integrative Epidemiology Unit at the University of Bristol, Bristol, United Kingdom; 3 Population Health Sciences, Bristol Medical School, Bristol, United Kingdom; Banaras Hindu University, INDIA

## Abstract

**Background:**

Mitochondria are organelles responsible for converting glucose into energy. Mitochondrial DNA is exclusively maternally inherited. The role of mitochondrial DNA haplogroups in the aetiology of cardiometabolic disease risk is not well understood.

**Methods:**

Sex-specific associations between common European mitochondrial DNA haplogroups (H, U, J, T, K, V, W, I and X) and trajectories of cardiometabolic risk factors from birth to 18 years were examined in a prospective cohort. Cardiometabolic risk factors measured from birth/mid-childhood to 18 years included body mass index (BMI), fat and lean mass, systolic and diastolic blood pressure, pulse rate, high-density lipoprotein cholesterol (HDL-c), non-HDL-c and triglycerides. Fractional polynomial and linear spline multilevel models explored the sex-specific association between haplogroups and risk factor trajectories.

**Results:**

Among a total of 7,954 participants with 79,178 repeated measures per outcome, we found no evidence that haplogroups U, T, J, K and W were associated with cardiometabolic risk factors compared to haplogroup H. In females, haplogroup V was associated with 4.0% (99% CI: -7.5, -0.6) lower BMI at age one but associations did not persist at age 18. Haplogroup X was associated with 1.3kg (99% CI: -2.5, -0.2) lower lean mass at age 9 which persisted at 18. Haplogroup V and X were associated with 9.3% (99% CI: -0.4, 19.0) and 16.4% (99% CI: -0.5,33.3) lower fat mass at age 9, respectively, although confidence intervals spanned the null and associations did not persist at 18. In males, haplogroup I was associated with 2.4% (99% CI: -0.5, 5.3) higher BMI at age 7; widening to 5.1% (99% CI: -0.5, 10.6) at 18 with confidence intervals spanning the null.

**Conclusions:**

Our study demonstrated little evidence of sex-specific associations between mitochondrial DNA haplogroups and cardiometabolic risk factors.

## Introduction

Mitochondria are organelles responsible for converting glucose into adenosine triphosphate, a form of energy that our cells are able to use [1, 2]. This process requires proteins, a small number of which are coded for by circular mitochondrial deoxyribose nucleic acid (DNA). Unlike nuclear DNA, mitochondrial DNA does not recombine and is exclusively maternally inherited by offspring [3]. Mutations throughout human history resulted in subdivisions of mitochondrial DNA into discrete region-specific haplogroups [4]. For instance, in Europe, 90% of the population belong to one of five major haplogroups. Variation in mitochondrial DNA may play a role in the aetiology of numerous diseases [5], including cardiometabolic disease [2] and cancer [6] as well as longevity [7], through differential production of reactive oxygen species that may impact oxidative stress of cells [2, 3, 7].

Evidence to date on the association between mitochondrial DNA haplogroups and cardiometabolic risk is conflicting [8–12], possibly influenced by variation in the interplay between mitochondrial and nuclear DNA-encoded, and environmental factors across populations [12, 13]. In a case-control study of 3,889 participants in the UK, haplogroup K was associated with risk of ischemic vascular events [11]. A prospective cohort study of 3,288 participants in the USA and a case-control study of 781 participants in Spain reported associations between haplogroup J and lower risk of ischemic cardiomyopathy and cardiovascular disease (CVD) [8, 9]. In contrast, a large prospective population-based study of 9,254 participants in Denmark did not find any strong evidence of associations between common European haplogroups and risk of ischaemic CVD [10]. Evidence has also been inconsistent for associations with key cardiometabolic risk factors. For instance, haplogroup X was associated with lower body mass index (BMI) and fat mass in a cross-sectional study of 2,286 adults in the US and haplogroup T was associated with higher risk of obesity in a case-control study of 716 adults in Southern Italy [14, 15]. Variations in mitochondrial DNA haplogroups have also been associated with obesity and metabolic syndrome is Asian populations [13, 16]. In contrast, a case-control study of 4,070 adults in Germany did not find evidence of associations between mitochondrial DNA haplogroups and obesity [17]. In addition, most studies have examined associations in adults only [8–11, 14, 15, 18], despite the early life origins of cardiometabolic risk which track through the life course [19, 20]. Furthermore, studies to date have not examined the sex-specific association of mitochondrial DNA with cardiometabolic risk factors [8–11, 14, 15, 17, 18, 21] although there are established sex differences in cardiometabolic risk across the life course [22–24]. Along with environmental factors associated with the development of childhood and adulthood obesity, a number of studies have identified obesity susceptible genes as responsible for the development of obesity [25, 26]. Given the complex aetiology of cardiometabolic disease further research is required to better understand the genetic predisposition to obesity and subsequent potential gene-environment interactions with the aim of developing effective prevention and treatment interventions.

Using data from a contemporary prospective birth cohort study in Southwest England, we examined the sex-specific associations between common European mitochondrial DNA haplogroups and cardiometabolic risk factors during childhood and adolescence. Risk factors included BMI (one to 18 years), height-adjusted fat and lean mass (9 to 18 years), systolic blood pressure (SBP), diastolic blood pressure (DBP) and pulse rate (7 to 18 years), high density lipoprotein cholesterol (HDL-c), non-high density lipoprotein cholesterol (non-HDL-c) and triglycerides (birth to 18 years).

## Materials and methods

### Study participants

The Avon Longitudinal Study of Parents and Children (ALSPAC) is a prospective birth cohort study in Southwest England [27, 28]. Pregnant women resident in Avon, UK with expected dates of delivery 1st April 1991 to 31st December 1992 were invited to take part in the study. The initial number of pregnancies enrolled is 14,541 (for these at least one questionnaire has been returned or a “Children in Focus” clinic had been attended by 19/07/99). Of these initial pregnancies, there was a total of 14,676 foetuses, resulting in 14,062 live births and 13,988 children who were alive at 1 year of age. When the oldest children were approximately 7 years of age, an attempt was made to bolster the initial sample with eligible cases who had failed to join the study originally. Therefore, the total sample size for analyses using any data collected after the age of 7 is 15,454 pregnancies, resulting in 15,589 foetuses. Of these 14,901 were alive at 1 year of age. The study has been described elsewhere in detail [27, 28]. Follow-up includes parent and child completed questionnaires, links to routine data and clinic attendance. Research clinics were held when the children were aged approximately 7, 9, 10, 11, 13, 15 and 18 years old. Ethical approval for the study was obtained from the ALSPAC Ethics and Law Committee and the Local Research Ethics Committees. This study used secondary data from the ALSPAC cohort. Participants in the original cohort study provided informed consent to participate and for their data to be used as secondary data in subsequent research following the recommendations of the ALSPAC Ethics and Law Committee at the time. The study website contains details of all the data that is available through a fully searchable data dictionary http://www.bristol.ac.uk/alspac/researchers/our-data/.

### Mitochondrial DNA haplogroup derivation

Children had DNA sampled at either birth from cord blood or at a research clinic at age 7. A total of 9,912 participants were genotyped using the Illumina HumanHap550 quad genome-wide SNP genotyping platform by Sample Logistics and Genotyping Facilities at the Wellcome Trust Sanger Institute and LabCorp (Laboratory Corporation of America) using support from 23andMe. The resulting raw genome-wide data were subjected to standard quality control (QC) methods. QC procedures removed related individuals, individuals of non-European genetic ancestry and individuals who withdrew consent. Population stratification was assessed by multidimensional scaling analysis and compared with Hapmap II (release 22) European descent (CEU), Han Chinese, Japanese and Yoruba reference populations; all individuals with non-European ancestry were removed. EIGENSTRAT analysis revealed no additional obvious population stratification and genome-wide analyses with other phenotypes indicate a low lambda). SNPs with a minor allele frequency of <1% and call rate of <95% or evidence for violations of Hardy-Weinberg equilibrium (P < 5E-7) were removed. After QC, 8,365 unrelated individuals were available for analysis. 7,554 custom mitochondrial probes, targeting 2,824 unique mitochondrial DNA positions, were included on the Illumina HumanHap550 quad genome-wide SNP genotyping platform. All heterozygous genotype calls (i.e. heteroplasmy) were set to missing prior to quality control using PLINK [29]. Genotype calls obtained from each probe were compared to the human mitochondrial database of non-pathological mitochondrial sequence variants (www.hmtdb.uniba.it:8080/hmdb/) to ensure that known allelic variants were being called. Haplogroup assignment was performed as described by Kloss-Brandstatter et al, using HaploGrep [30]. HaploGrep is a reliable algorithm for the automatic classification of mitochondrial DNA haplogroups that uses the latest version of Phylotree (http://www.phylotree.org/tree/index.htm). Samples with a quality score of more than 90% were used for our analysis [30]. Major haplogroups were defined as containing multiple haplogroups that are closely related to utilize information on less common haplogroups. Our analysis included nine common European haplogroup categories, H, U, T, J, K, V, I, W and X. Haplogroup HV was grouped with haplogroup V. Individuals with rare European and non-European haplogroups were excluded (A, C, D, L, M, N, R; n = 107). Further information on how haplogroups were derived including the frequency of each haplogroup are included in S1 Appendix and S1 Table in S1 File. S1 Fig in S1 File shows a flow diagram of participants eligible for inclusion.

### Cardiometabolic risk factor measurement

#### BMI, fat and lean mass

Length (before the age of 2 years), height (from the age of 2 years) and weight data from the age of 1 year were obtained from several sources including health visitor records, questionnaires and clinics from birth to 18 years. BMI was calculated as weight (kg) divided by height squared (m^2^). Whole body less head, and central fat and lean mass were derived from whole body dual energy X-ray absorptiometry (DXA) scans assessed five times at ages 9, 11, 13, 15, and 18 using a Lunar prodigy narrow fan beam densitometer.

#### SBP, DBP and pulse rate

At each clinic (ages 7, 9, 10, 11, 12, 15 and 18), SBP, DBP and pulse rate were measured at least twice in each with the child sitting and at rest with the arm supported, using a cuff size appropriate for the child’s upper arm circumference and a validated blood pressure monitor. The mean of the two final measures was used.

#### Blood based biomarkers

HDL-c, total cholesterol and triglycerides were measured in cord blood at birth and from venous blood subsequently. Samples were non-fasted at 7 and 9 years; fasting measures were available from clinics at 15 and 18 years. Non-HDL-c was calculated by subtracting HDL-c from total cholesterol at each measurement occasion. Trajectories of HDL-c, non-HDL-c and triglycerides were derived from a combination of measures from cord blood, non-fasting and fasting bloods.

Further information on details of measurement sources are included in S2 Appendix in S1 File.

### Statistical analysis

We used multilevel models to examine the association between mitochondrial DNA haplogroups and change in each risk factor across childhood and into adolescence. Multilevel models estimate mean trajectories of the risk factor while accounting for the non-independence (i.e. clustering) of repeated measurements within individuals, change in scale and variance of measures over time, and differences in the number and timing of measurements between individuals [31, 32]. We included all participants with at least one measure of the risk factor in each multilevel model, under a missing-at-random (MAR) assumption, to minimise selection bias [31, 32]. Sex-specific trajectories of all outcomes have been modelled previously using multilevel models and are described elsewhere in detail [22, 33–35]. Briefly, trajectories of BMI were modelled using fractional polynomials while trajectories of all other risk factors (fat mass, lean mass, SBP, DBP, pulse rate, HDL-c, non-HDL-c, triglycerides) were estimated using linear spline multilevel models (all models had two levels: measurement occasion and individual). Model fit statistics for each risk factor trajectory in our sample are shown in S2-S8 Tables in S1 File.

#### Association between mitochondrial DNA haplogroups and trajectories

Participants with data on sex, mitochondrial DNA haplogroup and at least one measure of a risk factor were included in analyses. Those who reported being pregnant at the 18-year clinic (n = 6) were excluded from analyses at that time point only. To explore the sex-specific association of mitochondrial DNA haplogroups with the above sex-specific trajectories of cardiometabolic risk factors, males and females were analysed separately. In all analyses, haplogroup H as the most common in the ALSPAC sample and in European populations was selected as the reference haplogroup. An interaction between each of the other haplogroups (U, T, J, K, V, W, I, X) and both the intercept and each fractional polynomial term (BMI) or spline (all other risk factors) was included in the models to estimate the difference in intercepts and slopes of each cardiometabolic risk factor between these haplogroups and the reference haplogroup, haplogroup H. All trajectories were modelled in MLwiN version 3.04, called from Stata version 16 using the runmlwin command [36].

In all models, age (in years) was centred at the first available measure. Values of cardiometabolic risk factors that had a skewed distribution (BMI, fat mass, triglycerides) were (natural) log transformed prior to analyses. Differences and confidence intervals for BMI, fat mass and triglycerides are therefore estimated on the log-scale. To aid interpretation, these estimates were back-transformed representing the ratio of geometric means and converted to percentage change post-analysis for simplicity of interpretation.

## Results

Table 1; S9 Table in S1 File shows the frequency of each haplogroup analysed in the study. Haplogroup H was the most frequent in the population, representing approximately 45% of males and females. The number of participants included in the analyses ranged from a total of 3,216 females (11,909 measures) and 3,144 males (10,955 measures) for fat mass, up to 3,886 females and 4,068 males for BMI (total measures of 39,778 and 39,400 respectively) (S10 Table, S1 Fig in S1 File). Compared with participants excluded from analyses, those included tended to have higher maternal education, higher household social class and lower maternal smoking during pregnancy (S11 Table in S1 File).

**Table 1:** Characteristics of ALSPAC participants included in analysis, by sex*.

	Female	Male
	n (%)	n (%)
**Haplogroup**		
H	1759 (45.3)	1818 (44.7)
U	515 (13.3)	548 (13.5)
T	401 (10.3)	415 (10.2)
J	420 (10.8)	448 (11.0)
K	300 (7.7)	390 (9.6)
V	243 (6.3)	205 (5.0)
W	67 (1.7)	80 (2.0)
I	125 (3.2)	101 (2.5)
X	56 (1.4)	63 (1.5)

*Number of participants available for analyses of BMI (n = 7,954) used as the denominator in this table given the varying sample sizes included in analyses.

### BMI, fat and lean mass

**Fig 1:**
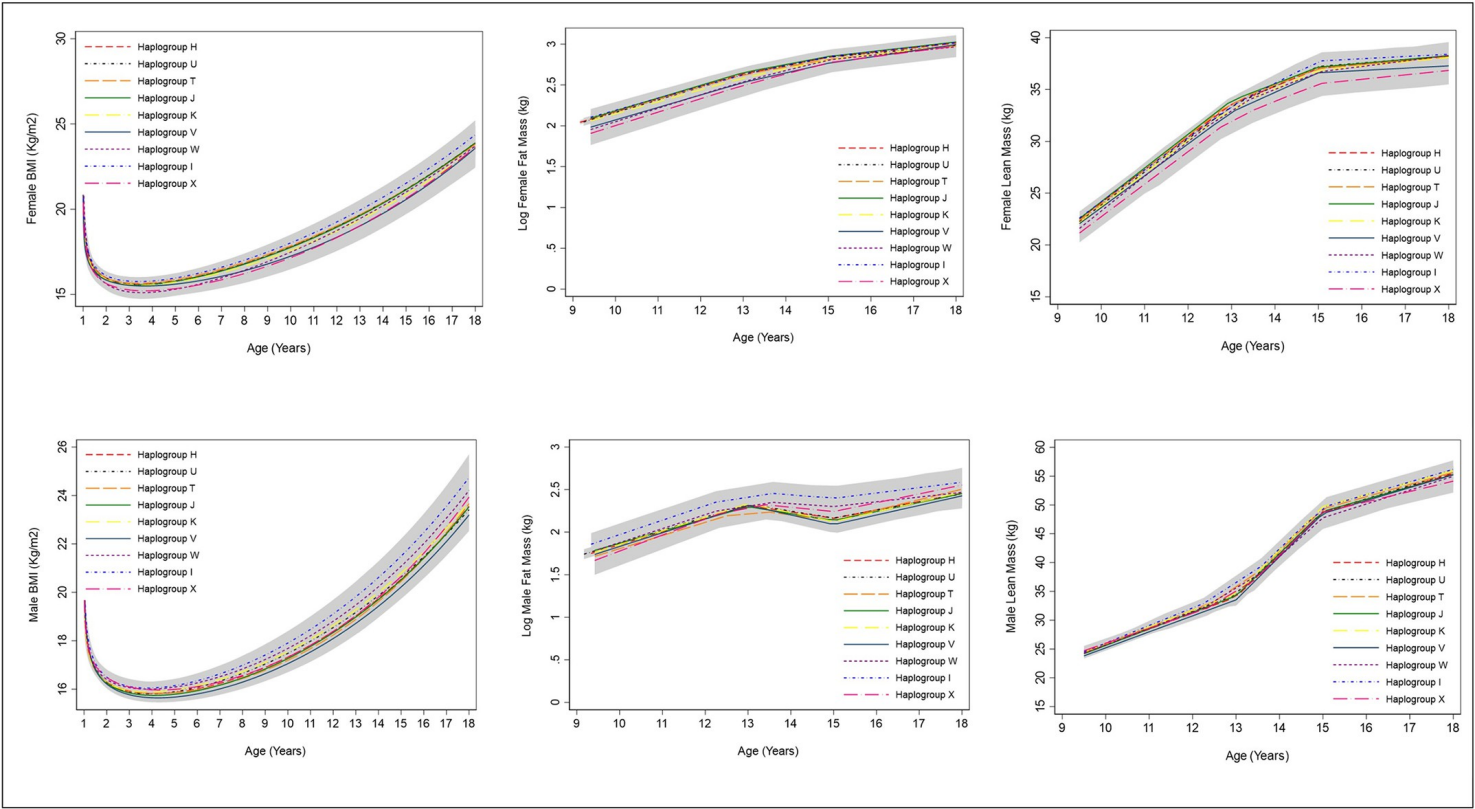
Mean trajectories of BMI, log fat mass and lean mass in females and males, by haplogroup. 99% confidence intervals for all haplogroups are displayed in grey. Note the different age range on the X axis for each outcome. Detailed results with confidence intervals are provided in S12-S14 Tables in S1 File. BMI, body mass index.

Among females, haplogroups U,T,J,K and I were not strongly associated with trajectories of BMI from one to 18 years and trajectories of fat mass and lean mass from 9 to 18 years. (Fig 1, S12-S14 Tables in S1 File). Haplogroup V was associated with 4.0% (99% confidence interval (CI): -7.5, -0.55) lower BMI at age 1 years, though this difference did not persist at age 18 (difference; -1.3%; 99% CI: -4.8, 2.2). Haplogroup V was associated with 9.3% (99% CI: -19.0, 0.3) lower fat mass at age 9 years, although the confidence intervals spanned the null; this difference reversed over childhood and adolescence, such that by age 18, haplogroup V was associated with -1.0 kg (99% CI: -1.9, -0.02) lower lean mass. Haplogroup X was associated with 16.4% (99% CI: -0.5, 33.3) lower fat mass at age 9 although the confidence interval spanned the null and this difference did not persist at age 18 (difference; 8.9% (99% CI: -19.6, 37.4)) (Fig 1, S13 Table in S1 File). Haplogroup W and X were associated with lower lean mass at age 9; this association persisted at age 18 for haplogroup X (difference; -1.4kg, 99% CI: -3.2, 0.4) but not for haplogroup W (difference; 0.04kg, 99% CI: -1.4, 1.8) (Fig 1, S14 Table in S1 File).

Among males, haplogroup I was associated with 2.4% (99% CI: -0.4, 5.3) higher BMI at 7 years; this increased to a difference of 5.1% (99%CI: -0.5, 10.6) at 18 years although confidence intervals spanned the null. Haplogroup I was also associated with higher fat mass in males at age 18 years only, albeit with wide confidence intervals spanning the null (difference; 31.8% (99% CI: -9.6, 73.2)). Haplogroup X was associated with higher fat mass at age 18, although also with wide confidence intervals spanning the null (37.7%, 99% CI: -17.4, 92.9).

### SBP, DBP, pulse

**Fig 2:**
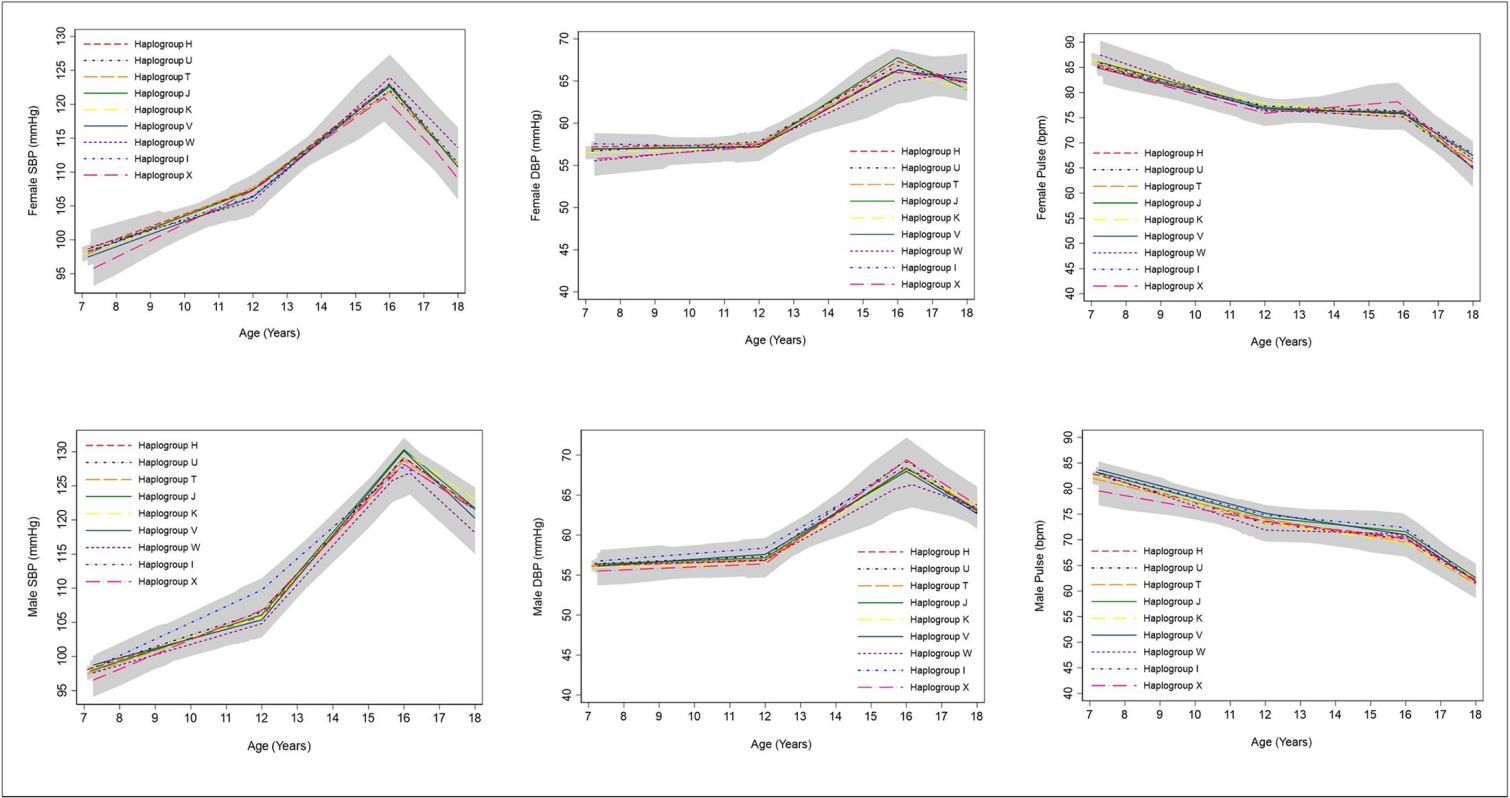
Mean trajectories of SBP, DBP and pulse in females and males, by haplogroup. 99% confidence intervals for all haplogroups are displayed in grey. Detailed results with confidence intervals are provided in S15 Table in S1 File. SBP, systolic blood pressure; DBP, diastolic blood pressure.

We found no strong evidence of associations between any haplogroup and trajectories of SBP, DBP or pulse from ages 7 to 18 years in females and males (Fig 2, S15 Table in S1 File).

### Blood based biomarkers

**Fig 3:**
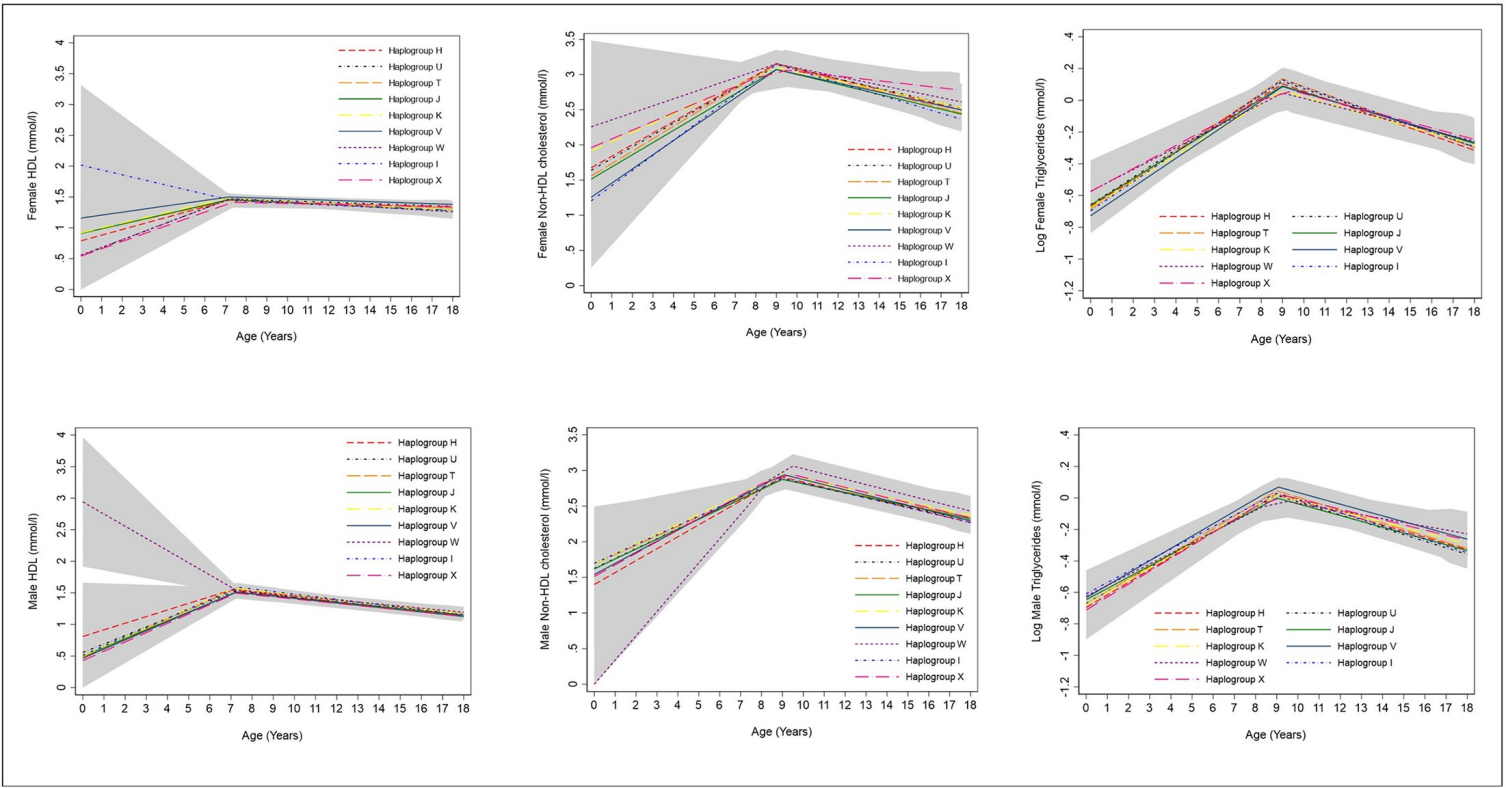
Mean trajectories of HDL-c, Non-HDL-c and log triglycerides in females and males, by haplogroup. 99% confidence intervals for all haplogroups are displayed in grey. Detailed results with confidence intervals are provided in S16 & S17 Tables in S1 File. Note the different age range on the X axis for each outcome. HDL-c, high density lipoprotein cholesterol.

We found no strong evidence of associations between haplogroups U,T,J,K,V,I and X and trajectories of HDL-c and non-HDL-c from birth to 18 years in females and males in comparison with haplogroup H (Fig 3, S16 Table in S1 File). Haplogroup W was associated with 2.1 mmol/l (99% CI: 0.8, 3.5) higher HDL-c at birth in males and 1.5mmol/l (99% CI: 0.4, 2.7) lower non-HDL-c but this difference did not persist at age 18 years (HDL difference: 0.05mmol/l (99% CI: -0.1, 0.2); non-HDL difference: 0.2mmol/l (99% CI: -0.1,0.5)). We found no strong evidence of associations between haplogroups and triglyceride trajectories of females and males from birth to 18 years (Fig 3, S17 Table in S1 File).

## Discussion

In this large prospective cohort study, we examined the sex-specific association between mitochondrial DNA haplogroups and longitudinal changes in nine cardiometabolic risk factors from birth or early childhood through to 18 years. We found no strong evidence of associations between major mitochondrial DNA haplogroups and trajectories of cardiometabolic risk factors. There was limited evidence of sex-specific associations between mitochondrial DNA haplogroups V, I and X and trajectories of adiposity.

### Comparison with other studies

Most studies to date have examined the association between mitochondrial DNA and cardiometabolic risk factors in adults and have not explored the contribution of mitochondrial DNA to the sex-specific aetiology of cardiometabolic risk across the early life course [8, 10, 18]. However, our overall findings are comparable with some previous analyses of females and males combined. For instance, a previous population-based study of 9,254 adults in Denmark [10] did not find evidence of associations between mitochondrial DNA haplogroups and cardiometabolic risk factors. Our results are also largely similar to two case-control studies comparing those with obesity to those without [17, 37], which found no evidence of associations of obesity with any haplogroup. We did however find some limited evidence that haplogroup I among males was associated with higher BMI from age 7 to age 18 years and higher fat mass at 18 years, albeit with confidence intervals spanning the null. This is consistent with findings in a prospective study of 2,342 participants aged 45 to 79 years recruited from four clinical sites in the US which showed increased incidence of obesity in males and females with haplogroup I, W and X combined [18]. Our findings build on this evidence, indicating that the observed increased BMI associated with haplogroup I, X and W combined in that study may be driven predominantly by haplogroup I, is potentially driven by fat mass rather than lean mass, may be unique to males and emerge in childhood, potentially tracking through to adulthood. Similarly, our findings of lower fat mass at age 9 and lower lean mass from age 9 to 18 years in females with haplogroup X are consistent with previous findings in a sex-combined analysis of 2,286 Caucasian adults of Northern European origin living in the US with a mean age of 51 years [15] which found lower BMI and fat mass in adults with haplogroup X. Our results indicate that associations of haplogroup X with BMI may be specific to females, are potentially driven by associations with both fat and lean mass and potentially begin in early life.

In contrast, we found no strong evidence of an association of haplogroup T and BMI or fat mass, as found in two case-control studies, one conducted in Southern Italy with 716 adults [14] and another Austrian study with 514 juveniles (aged less than 21 years) and 1,598 adults [21]. However, we did find evidence of an association between haplogroup W and HDL-c at birth in males which to our knowledge has not been demonstrated previously. Reasons for differences in findings between our work and previous studies may include the examination of mitochondrial DNA haplogroups and outcomes in both sexes combined in previous studies, thus potentially overlooking the sex-specific aetiology of mitochondrial DNA haplogroups and cardiometabolic risk examined here. A further possibility for differences between our study and previous work may include our examination of trajectories of repeated continuous measures of risk factors from birth/early childhood to 18 years, allowing us to examine when associations emerge and whether they persist over time in females and males. Further work is required to extend such analyses beyond 18 years to examine if mitochondrial DNA haplogroups are associated with changes in risk factors from early and mid-adulthood into older age. Variability across studies may be explained by variation in the nuclear genetic background of study populations [13, 38]. Further, mitochondrial DNA sequence diversity varies greatly across populations [12]. Co-evolution and the complex interaction between mitochondrial and nuclear genomes make it difficult to compare results across study populations [38]. The variation in the interplay between mitochondrial and nuclear DNA-encoded, and environmental factors across populations may explain the observed differences [38, 39]. For instance, in one study exploring the association between mitochondrial genetic factors and type 2 diabetes, associations were only observed in specific mitonuclear genotype combinations. In addition, the interplay with environmental factors and ethnicity may also be important in explaining variation across populations [40, 41].

Cardiometabolic diseases are complex conditions in which oxidative phosphorylation may play a role. Variation in mitochondrial DNA may lead to differential production of reactive oxygen species that impacts the oxidative stress of cells [2, 3, 7]. However, the majority of polymorphisms defining the mitochondrial haplogroups are synonymous. Mitochondrial DNA haplogroups differ in the average number of nonsynonymous mutations and in the nonsynonymous to synonymous rate ratio [42]. For instance, on average a 2.5-fold higher number of nonsynonymous differences are observed in the haplogroup cluster IWX compared to other European haplogroups which may contribute to lower complex I activity. However, observed associations between haplogroups and cardiometabolic risk factors require further exploration to determine whether causation exists. The majority of proteins are encoded by the nuclear genome with 13 originating from mitochondrial DNA [43], which alone are not sufficient to drive mitochondrial activities [12]. A complex interplay exists between mitochondrial and nuclear DNA-encoded and environmental factors [13]. In particular, evidence suggests a link between mitonuclear interactions within complex I and cardiometabolic disease [39]. Further developments in methods and techniques to directly test the causative role of mitochondrial DNA in disease aetiology are required [5]. For instance, a recent study, deep sequencing the whole mitochondrial DNA molecule in 5,491 individuals, demonstrated that mitochondrial DNA heteroplasmy may contribute to the pathogenesis of hypertension via the mitochondrial respiratory chain function [44].

### Strengths and limitations

There are a number of strengths to our study including the availability of repeated measures of cardiometabolic risk factors throughout from birth/early to age 18 years and the use of multilevel models allowing for clustering of repeated measures within individuals and correlation between measures over time. Due to the large sample size, we were able to examine rarer haplogroups such as W, I and X separately. However, our data would not allow for a more fine-scaled haplogroup classification and we were unable to explore whether associations varied by mutations within major mitochondrial DNA haplogroups. In addition, we have conducted sex-specific analyses to account for the differences in risk factor trajectories previously reported [22] and to better understand the associations between mitochondrial DNA haplogroups and cardiometabolic risk factors in females and males. The study also has some limitations. Due to sparsity of measures, we were only able to explore linear change over time. Loss-to-follow up is another potential limitation of our study. However, we have included all participants with at least one measure of each risk factor to minimise any potential selection bias. The number of people with measurements of each risk factor also varied meaning that our sample sizes differed for each risk factor and thus are not directly comparable. Our sample may be underpowered to detect effects for rarer haplogroups and additionally some observed associations may be a result of type 1 error due to multiple testing.

## Conclusion

Our study found no strong evidence of associations between major mitochondrial DNA haplogroups and trajectories of cardiometabolic risk factors during childhood and adolescence. There was some limited evidence of sex-specific associations between mitochondrial DNA haplogroups V, I and X and trajectories of adiposity.

## Supporting information

S1 File (DOCX)
