# Supplementary material for: Mitochondrial DNA haplogroups and trajectories of cardiometabolic risk factors during childhood and adolescence: A prospective cohort study
Source: PLoS One. 2023 Apr 12;18(4):e0284226. doi: 10.1371/journal.pone.0284226 (PMC10096512; doi:10.1371/journal.pone.0284226)
Supplement: S1 File — (DOCX) [file pone.0284226.s001.docx]

**Mitochondrial DNA haplogroups and trajectories of cardiometabolic risk factors during childhood and adolescence: a prospective cohort study**

**Supplementary Material**

Kate N O’Neill ^1^, PhD; Emily Aubrey ^2, 3^ MB ChB; Laura D Howe ^2, 3^, PhD; Evie Stergiakouli ^2,3^, PhD; Santiago Rodriguez ^2,3^, PhD; Patricia M Kearney ^1^ , PhD; Linda M O’Keeffe ^1, 2, 3^, PhD

^1^ School of Public Health, University College Cork, Ireland

^2^ MRC Integrative Epidemiology Unit at the University of Bristol, Bristol, UK

^3^ Population Health Sciences, Bristol Medical School Bristol, UK

**Corresponding author:** Dr Kate O’Neill, School of Public Health, 4^th^ Floor Western Gateway Building, University College Cork, Ireland

**Email**

[Kate.oneill@ucc.ie](mailto:Kate.oneill@ucc.ie)

**Supplementary Material Contents**

**Appendix S1:** Mitochondrial DNA haplogroup derivation

**Figure S1:** Flow diagram of study

**Appendix S2:** Details of measurement sources

**Table S1:** Frequency of Mitochondrial DNA Haplogroups in full sample

**Table S2:** Model details for log BMI trajectories

**Table S3:** Model details for log fat mass trajectories

**Table S4:** Model details for lean mass trajectories

**Table S5:** Model details for SBP, DBP and pulse rate trajectories

**Table S6:** Model details for HDL-c

**Table S7:** Model details for non-HDL-c trajectories

**Table S8:** Model details for log triglyceride trajectories

**Table S9**: Frequencies of haplogroups among participants with at least 1 measure of each risk factor

**Table S10:** Number of participants with cardiometabolic risk factor measures at each time point

**Table S11:** Characteristics at birth of the mothers of children included in models compared with those excluded due to missing exposure or outcome data

**Table S12:** Mean trajectories of BMI estimated from multilevel models, by haplogroup

**Table S13**: Mean trajectories of fat mass estimated from multilevel models, by haplogroup

**Table S14:** Mean trajectories of lean mass estimated from multilevel models, by haplogroup

**Table S15:** Mean trajectories of SBP, DBP and pulse rate estimated from multilevel models, by haplogroup

**Table S16:** Mean trajectories of HDL-c and non-HDL-c estimated from multilevel models**,** by haplogroup

**Table S17:** Mean trajectories of triglyceride estimated from multilevel models**,** by haplogroup

**Appendix S1:** Mitochondrial Haplogroup derivation

A total of 9,912 participants were genotyped using the Illumina HumanHap550 quad genome-wide SNP genotyping platform by Sample Logistics and Genotyping Facilities at the Wellcome Trust Sanger Institute and LabCorp (Laboratory Corporation of America) using support from 23andMe. The resulting raw genome-wide data were subjected to standard quality control (QC) methods. Individuals were excluded from further analysis on the basis of having incorrect gender assignments, minimal or excessive heterozygosity (<0.320 and >0.345 for the Sanger data and <0.310 and >0.330 for the LabCorp data), disproportionate levels of individual missingness (>3%), evidence of cryptic relatedness (>10% IBD) and insufficient sample replication (IBD < 0.8). Population stratification was assessed by multidimensional scaling analysis and compared with Hapmap II (release 22) European descent (CEU), Han Chinese, Japanese and Yoruba reference populations; all individuals with non-European ancestry were removed. EIGENSTRAT analysis revealed no additional obvious population stratification and genome-wide analyses with other phenotypes indicate a low lambda). SNPs with a minor allele frequency of <1% and call rate of <95% or evidence for violations of Hardy-Weinberg equilibrium (P < 5E-7) were removed. After QC, 8,365 unrelated individuals were available for analysis. 7,554 custom mitochondrial probes, targeting 2,824 unique mitochondrial DNA positions, were included on the Illumina HumanHap550 quad genome-wide SNP genotyping platform. All heterozygous genotype calls (i.e. heteroplasmy) were set to missing prior to quality control using PLINK (1). Genotype calls obtained from each probe were compared to the human mitochondrial database of non-pathological mitochondrial sequence variants (www.hmtdb.uniba.it:8080/hmdb/) to ensure that known allelic variants were being called. Probes were excluded in cases where genotype calls were not represented in the Cambridge Reference Sequence reference (rCRS) or one of the known allelic variants. Probes with an overall call rate of <95% were excluded prior to analysis. The genotyping concordance of the remaining probes was investigated by comparing the genotype calls in 445 replicate samples. With the exception of probe failure (i.e. missing data), a 100% genotyping concordance rate was obtained for each probe. All probes with a failure rate of >5% in the replicate sample were further excluded. In cases where multiple probes passed the above-mentioned QC criteria, the probe with the highest calling rate was used for analysis. A total of 1,062 probes passed QC for the batch that was genotyped by Laboratory Corporation of America (n=7,590), whilst 629 probes passed QC for the batch genotyped by the Sanger Institute (n=775). 308 of these probes overlapped with each other across the two arrays. Where there were several probes genotyping the same mitochondrial DNA variant, we used a consensus of the probes and removed all duplicates. This resulted in 105 unique variants, all with MAF>0.01 (29 with MAF>0.05). Haplogroup assignment was performed as described by Kloss-Brandstatter et al, using HaploGrep (2). HaploGrep is a reliable algorithm for the automatic classification of mitochondrial DNA haplogroups that uses the latest version of Phylotree (http://www.phylotree.org/tree/index.htm). Samples with a quality score of more than 90% were used for our analysis (28). Major haplogroups were defined as containing multiple haplogroups that are closely related to utilize information on less common haplogroups. After QC and removing individuals with withdrawn consent; our dataset contained 8,209 individuals with derived mitochondrial DNA haplogroups. Our analysis included nine common European haplogroup categories, H, U, T, J, K, V, I, W and X. Individuals with rare European and non-European haplogroups were excluded from our analysis (A, C, D, L, M, N, R; n=107). Figure S1 shows a flow diagram of the study sample.

**Figure S1: Flow diagram of study**


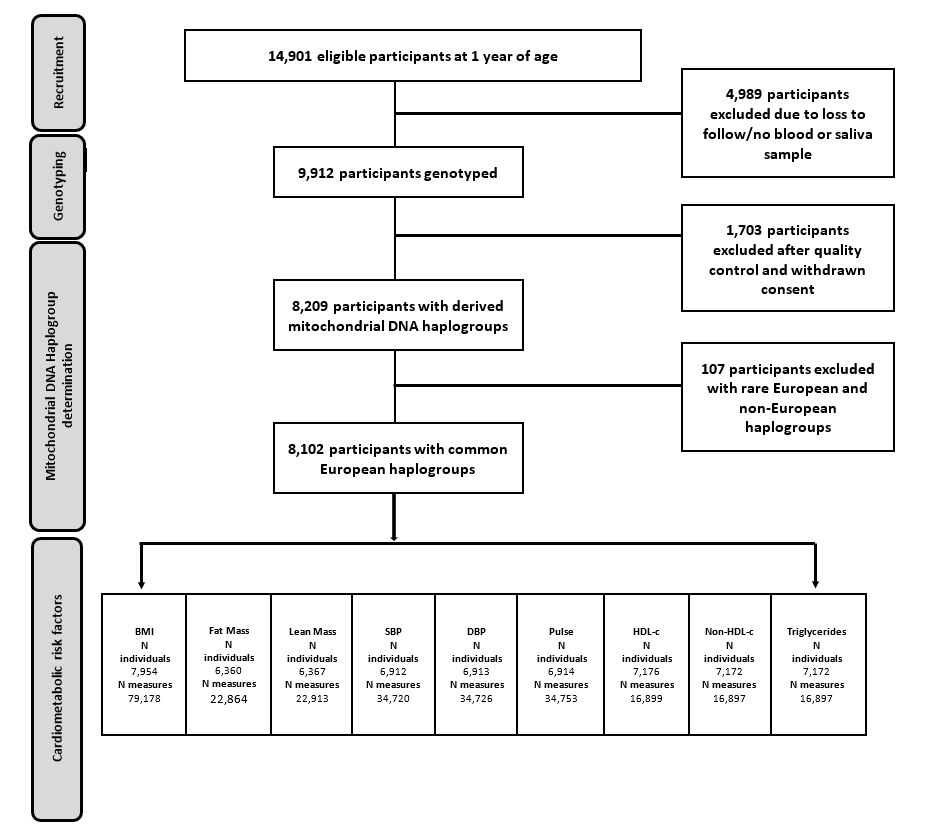


BMI, body mass index; SBP, systolic blood pressure; DBP, diastolic blood pressure; HDL-c, high density lipoprotein cholesterol.

**Appendix S2:** Details of measurement sources

**Details of measurement sources**

*Details on measurement of anthropometry*

Data from age 1 onwards are included in this analysis. We did not include measures before 1 year because of the difficulty in accurately modelling BMI from birth through the whole of childhood due to its early peak followed by adiposity rebound. From 1 to 5 years, measures were available from routine child health clinics for most children and extracted from health visitor records, which form part of standard child care in the UK. Data were also available from research clinic measurements on a random 10% subsample of the cohort. All cohort members were invited to research clinics from age 7 onwards. Across all ages parent-reported measures were available.

At the clinics, crown-heel length for children aged four to 25 months was measured using a Harpenden Neonatometer and from 25 months onwards standing height was measured using a Leicester Height Measure; weight was measured using Fereday 100kg combined scale (four-month clinic), Soenhle scale or Seca scale model 724 (eight-month clinic), Seca 724 or Seca 835 (12-month clinic), Seca 835 (18 months onwards). From age 7 years, all children were invited to annual clinics, at which standing height was measured to the last complete mm using the Harpenden Stadiometer and weight was measured to the nearest 0.1kg using the Tanita Body Fat Analyser (Model TBF 305).

*Details on measurement of blood pressure*

A Dinamap 9301 Vital Signs Monitor (Morton Medical, London) was used at the 7, 9, and 11-year clinics; an Omron MI-5 was used at the 10-year clinic; a Dinamap 8100 Vital Signs Monitor (Morton Medical) was used at the 13-year clinic; and an Omron IntelliSense M6 (Omron Healthcare, Kyoto, Japan) was used at the 15- and 18-year clinics.

*Details on measurement of blood-based biomarkers*

Plasma lipid assays (triglycerides and high-density lipoprotein cholesterol (HDL-c)) were performed by modification of the standard Lipid Research Clinics Protocol using enzymatic reagents for lipid determination. All assay coefficients of variation were <5%.

| **Haplogroup** | **N = 8,209** | **%** |
| --- | --- | --- |
| **H** | 3,649 | 44.45 |
| **U** | 1,083 | 13.19 |
| **T** | 830 | 10.11 |
| **J** | 883 | 10.76 |
| **K** | 704 | 8.58 |
| **V** | 456 | 5.55 |
| **W** | 147 | 1.79 |
| **I** | 231 | 2.81 |
| **X** | 119 | 1.45 |
| **M** | 29 | 0.35 |
| **L** | 28 | 0.34 |
| **N** | 18 | 0.22 |
| **R** | 14 | 0.17 |
| **A** | 8 | 0.10 |
| **D** | 7 | 0.09 |
| **C** | 3 | 0.04 |

**Table S1:** Frequency of mitochondrial DNA haplogroups in full sample**Table S2:** Model details for log BMI trajectories

|  | Number of contributing individuals | | Assessment of model fit | | | | |
| --- | --- | --- | --- | --- | --- | --- | --- |
|  | Total observations | Individuals with 1 measure | Mean observed  BMI, ln(kg/m^2^) (SD) ^a^ | Mean predicted  BMI, ln(kg/m^2^) (SD) ^a^ | Mean difference (observed – predicted), ln(kg/m^2^) ^a^ | 95% level of agreement between observed and predicted, ln(kg/m^2^) ^a^ |  |
| Female |  |  |  |  |  |  |  |
| Overall | 39778 | 3886 |  |  |  |  |  |
| 1-3 years | 6307 | 3034 | 2.81 (0.09) | 2.81 (0.07) | 0.004 (0.07) | -0.09 to 0.10 |  |
| 3-7 years | 8328 | 3320 | 2.77 (0.11) | 2.77 (0.08) | -0.001 (0.08) | -0.14 to 0.14 |  |
| 7-9 years | 5272 | 3055 | 2.81 (0.13) | 2.82 (0.12) | -0.01 (0.12) | -0.08 to 0.06 |  |
| 9-11 years | 6078 | 3065 | 2.88 (0.16) | 2.88 (0.14) | -0.001 (0.14) | -0.08 to 0.08 |  |
| 11-13 years | 5018 | 2876 | 2.95 (0.17) | 2.94 (0.15) | 0.01 (0.15) | -0.07 to 0.10 |  |
| 13-15 years | 4610 | 2754 | 3.00 (0.16) | 2.99 (0.15) | 0.01 (0.15) | -0.11 to 0.12 |  |
| 15-18 years | 4165 | 2478 | 3.08 (0.15) | 3.10 (0.15) | -0.01 (0.15) | -0.10 to 0.08 |  |
| Male |  |  |  |  |  |  |  |
| Overall | 39400 | 4068 |  |  |  |  |  |
| 1-3 years | 6653 | 3207 | 2.84 (0.09) | 2.83 (0.07) | 0.01 (0.07) | -0.09 to 0.10 |  |
| 3-7 years | 8811 | 3525 | 2.78 (0.10) | 2.78 (0.07) | -0.002 (0.07) | -0.13 to 0.12 |  |
| 7-9 years | 5297 | 3104 | 2.79 (0.12) | 2.80 (0.11) | -0.01 (0.11) | -0.08 to 0.06 |  |
| 9-11 years | 5978 | 3007 | 2.86 (0.15) | 2.86 (0.14) | 0.002 (0.14) | -0.07 to 0.07 |  |
| 11-13 years | 4666 | 2762 | 2.93 (0.16) | 2.92 (0.15) | 0.01 (0.15) | -0.06 to 0.09 |  |
| 13-15 years | 4441 | 2676 | 2.97 (0.16) | 2.97 (0.15) | 0.001 (0.15) | -0.10 to 0.10 |  |
| 15-18 years | 3554 | 2181 | 3.06 (0.15) | 3.07 (0.15) | -0.01 (0.15) | -0.09 to 0.07 |  |

SD, standard deviation; ln(kg/m^2^), natural log of kilograms per metre squared

^a^ BMI is presented in the natural log and values represent the mean predicted natural log of BMI at each age shown.

**Table S3:** Model details for log fat mass trajectories

|  | **Number of contributing individuals** | | **Assessment of model fit** | | | |
| --- | --- | --- | --- | --- | --- | --- |
|  | Total observations | Individuals with 1 measure | Mean observed, ln(kg) (SD)^a^ | Mean predicted, ln(kg) (SD)^aa^ | Mean difference (observed – predicted), ln(kg)^a^ | 95% level of agreement between observed and predicted, ln(kg)^a^ |
| **Female** |  |  |  |  |  |  |
| Overall | 11909 | 3216 |  |  |  |  |
| 9 years | 2803 | 2803 | 2.14 (0.50) | 2.14 (0.48) | -0.003 | -0.15 to 0.14 |
| 9-13 years | 5496 | 3083 | 2.28 (0.52) | 2.28 (0.49) | 0.002 | -0.17 to 0.17 |
| 13-15 years | 2394 | 2373 | 2.69 (0.44) | 2.70 (0.42) | -0.01 | -0.21 to 0.20 |
| 15-18 years | 4019 | 2421 | 2.91 (0.41) | 2.91 (0.38) | 0.002 | -0.16 to 0.16 |
| **Male** |  |  |  |  |  |  |
| Overall | 10955 | 3144 |  |  |  |  |
| 9 years | 2705 | 2705 | 1.81 (0.59) | 1.83 (0.55) | -0.02 | -0.23 to 0.20 |
| 9-13 years | 5274 | 3009 | 1.98 (0.62) | 1.97 (0.57) | 0.01 | -0.23 to 0.26 |
| 13-15 years | 2238 | 2228 | 2.20 (0.62) | 2.25 (0.57) | -0.05 | -0.36 to 0.27 |
| 15-18 years | 3443 | 2125 | 2.31 (0.64) | 2.30 (0.60) | 0.01 | -0.23 to 0.25 |

SD, standard deviation; ln(kg), natural log of kilograms

^a^Fat mass is presented in the natural log and values represent the mean predicted natural log of fat mass.

**Table S4:** Model details for lean mass trajectories

|  | **Number of contributing individuals** | | **Assessment of model fit** | | | | |
| --- | --- | --- | --- | --- | --- | --- | --- |
|  | **Total observations** | **Individuals with 1 measure** | **Mean observed, kg (SD)** | **Mean predicted, kg (SD)** | | **Mean difference (observed – predicted), kg** | **95% level of agreement between observed and predicted, kg** |
| **Female** |  |  |  |  |  | |  |
| Overall | 11940 | 3220 |  |  |  | |  |
| 9 years | 2807 | 2807 | 23.61 (3.11) | 23.55 (2.94) | 0.06 | | -2.28 to 2.40 |
| 9-13 years | 5506 | 3087 | 26.40 (4.74) | 26.46 (4.51) | -0.07 | | -2.38 to 2.25 |
| 13-15 years | 2402 | 2381 | 35.27 (4.00) | 35.01 (3.85) | 0.26 | | -2.06 to 2.58 |
| 15-18 years | 4032 | 2428 | 37.55 (4.06) | 37.62 (3.75) | -0.06 | | -2.05 to 1.93 |
| **Male** |  |  |  |  |  | |  |
| Overall | 10973 | 3147 |  |  |  | |  |
| 9 years | 2711 | 2711 | 25.54 (2.91) | 25.38 (2.39) | 0.16 | | -3.56 to 3.88 |
| 9-13 years | 5283 | 3013 | 27.80 (4.26) | 27.95 (4.21) | -0.14 | | -3.49 to 3.20 |
| 13-15 years | 2240 | 2230 | 40.95 (7.12) | 40.39 (6.08) | 0.55 | | -3.36 to 4.47 |
| 15-18 years | 3450 | 2129 | 52.24 (7.06) | 52.38 (6.69) | -0.14 | | -2.86 to 2.59 |

SD, standard deviation; kg, kilograms

**Table S5:** Model details for SBP, DBP and pulse rate trajectories

|  | Number of contributing individuals | | Assessment of model fit | | | | |
| --- | --- | --- | --- | --- | --- | --- | --- |
|  | Total observations | Individuals with 1 measure | Mean observed SBP, DBP or pulse rate (SD) ^a^ | Mean predicted SBP, DBP or pulse rate (SD) ^a^ | Mean difference (observed–predicted) ^a^ | | 95% level of agreement between observed and predicted ^a^ |
| SBP |  |  |  |  | |  |  |
| Females |  |  |  |  | |  |  |
| Overall | 17863 | 3453 |  |  | |  |  |
| 7 years | 2926 | 2926 | 98.95 (9.25) | 98.79 (5.57) | | 0.16 | -10.59 to 10.92 |
| 7-12 years | 11035 | 3373 | 102.88 (9.75) | 103.02 (6.34) | | -0.13 | -11.98 to 11.72 |
| 12-16 years | 4776 | 2768 | 114.66 (11.04) | 114.20 (7.79) | | 0.46 | -11.31 to 12.23 |
| 16-18 years | 2052 | 1986 | 112.54 (8.48) | 112.87 (5.44) | | -0.33 | -12.72 to 12.06 |
| Males |  |  |  |  | |  |  |
| Overall | 16857 | 3459 |  |  | |  |  |
| 7 years | 2989 | 2989 | 98.68 (9.04) | 98.61 (5.34) | | 0.08 | -10.43 to 10.58 |
| 7-12 years | 10801 | 3392 | 102.29 (9.34) | 102.41 (6.12) | | -0.12 | -11.47 to 11.23 |
| 12-16 years | 4450 | 2626 | 116.94 (12.41) | 116.53 (9.68) | | 0.40 | -11.46 to 12.27 |
| 16-18 years | 1606 | 1560 | 122.38 (9.48) | 122.67 (5.93) | | -0.29 | -12.07 to 11.48 |
| DBP  Females |  |  |  |  | |  |  |
| Overall | 17869 | 3454 |  |  | |  |  |
| 7 years | 2926 | 2926 | 56.69 (6.53) | 56.91 (3.37) | | -0.22 | -9.00 to 8.55 |
| 7-12 years | 11045 | 3373 | 58.45 (7.01) | 58.01 (3.71) | | 0.44 | -9.71 to 10.59 |
| 12-16 years | 4776 | 2768 | 60.97 (9.25) | 61.86 (4.70) | | -0.89 | -12.56 to 10.77 |
| 16-18 years  Males | 2048 | 1981 | 64.65 (5.87) | 64.94 (3.47) | | -0.29 | -13.13 to 12.55 |
| Overall | 16857 | 3459 |  |  | |  |  |
| 7 years | 2988 | 2988 | 55.94 (6.63) | 56.19 (3.43) | | -0.24 | -8.70 to 8.21 |
| 7-12 years | 10802 | 3392 | 57.60 (6.86) | 57.19 (3.56) | | 0.41 | -9.42 to 10.23 |
| 12-16 years | 4451 | 2626 | 61.35 (10.12) | 62.23 (5.67) | | -0.88 | -12.49 to 10.73 |
| 16-18 years | 1604 | 1559 | 63.44 (6.17) | 63.82 (3.72) | | -0.38 | -12.81 to 12.06 |
| Pulse  Females |  |  |  |  | |  |  |
| Overall | 17888 | 3455 |  |  | |  |  |
| 7 years | 2920 | 2927 | 84.31 (10.75) | 84.83 (5.74) | | -0.52 | -14.37 to 13.34 |
| 7-12 years | 10828 | 3373 | 79.69 (11.28) | 79.33 (7.19) | | 0.27 | -14.03 to 14.57 |
| 12-16 years | 4765 | 2769 | 75.80 (11.04) | 76.29 (6.30) | | -0.50 | -14.74 to 13.74 |
| 16-18 years | 2052 | 1993 | 67.58 (9.98) | 67.85 (5.90) | | -0.28 | -14.93 to 14.37 |
| Pulse  Males |  |  |  |  | |  |  |
| Overall | 16865 | 3459 |  |  | |  |  |
| 7 years | 2990 | 2991 | 81.68 (10.57) | 81.94 (6.03) | | -0.26 | -11.79 to 11.28 |
| 7-12 years | 10406 | 3392 | 76.20 (11.31) | 76.04 (7.63) | | 0.003 | -12.93 to 12.94 |
| 12-16 years | 4443 | 2626 | 72.15 (11.13) | 72.07 (6.67) | | 0.07 | -13.32 to 13.47 |
| 16-18 years | 1606 | 1561 | 63.07 (9.53) | 63.30 (5.38) | | -0.22 | -14.55 to 14.10 |

DBP, diastolic blood pressure; SBP, systolic blood pressure; SD, standard deviation

^a^units are presented in mmHg for SBP and DBP and bpm for pulse rate

**Table S6:** Model details for HDL-c trajectories

|  | **Number of contributing individuals** | | **Assessment of model fit** | | | |
| --- | --- | --- | --- | --- | --- | --- |
|  | Total observations | Individuals with 1 measure | Mean observed, mmol/l (SD) | Mean predicted, mmol/l (SD) | Mean difference (observed – predicted), mmol/l | 95% level of agreement between observed and predicted, mmol/l |
| **Females** |  |  |  |  |  |  |
| Overall | 8463 | 3533 |  |  |  |  |
| Birth | 1474 | 1474 | 0.84 (3.98) | 0.84 (3.97) | 0.00000002 | -0.02 to 0.02 |
| 0-7 years | 3488 | 2799 | 1.22 (2.62) | 1.20 (2.60) | 0.02 | -0.18 to 0.22 |
| 7-18 years | 6988 | 3099 | 1.40 (0.31) | 1.40 (0.22) | 0.0000 | -0.28 to 0.28 |
| **Male** |  |  |  |  |  |  |
| Overall | 8436 | 3643 |  |  |  |  |
| Birth | 1515 | 1515 | 0.69 (2.98) | 0.69 (2.97) | -0.000002 | -0.02 to 0.02 |
| 0-7 years | 3640 | 2969 | 1.19 (1.98) | 1.18 (1.97) | 0.005 | -0.17 to 0.18 |
| 7-18 years | 6920 | 3133 | 1.38 (0.32) | 1.38 (0.26) | -0.00002 | -0.25 to 0.25 |

HDL-c, high density lipoprotein cholesterol; SD, standard deviation; mmol/l, millimole per litre

**Table S7:** Model details for non-HDL-c trajectories

|  | **Number of contributing individuals** | | **Assessment of model fit** | | | |
| --- | --- | --- | --- | --- | --- | --- |
|  | Total observations | Individuals with 1 measure | Mean observed, mmol/l (SD) | Mean predicted, mmol/l (SD) | Mean difference (observed – predicted), mmol/l | 95% level of agreement between observed and predicted, mmol/l |
| **Females** |  |  |  |  |  |  |
| Overall | 8458 | 3529 |  |  |  |  |
| Birth | 1474 | 1474 | 1.07 (4.12) | 1.09 (4.00) | -0.02 | -0.26 to 0.21 |
| 0-7 years | 3575 | 2872 | 2.19 (2.85) | 2.15 (2.74) | 0.05 | -0.62 to 0.71 |
| 7-18 years | 4883 | 2645 | 2.74 (0.68) | 2.78 (0.51) | -0.03 | -0.81 to 0.74 |
| **Males** |  |  |  |  |  |  |
| Overall | 8439 | 3643 |  |  |  |  |
| Birth | 1515 | 1515 | 1.07 (3.11) | 1.08 (3.06) | -0.02 | -0.15 to 0.12 |
| 0-7 years | 3730 | 3040 | 2.08 (2.20) | 2.05 (2.13) | 0.03 | -0.37 to 0.44 |
| 7-18 years | 4709 | 2587 | 2.55 (0.64) | 2.58 (0.51) | -0.03 | -0.56 to 0.51 |

Non-HDL-c, non-high density lipoprotein cholesterol; SD, standard deviation; mmol/l, millimole per litre

**Table S8:** Model details for log triglyceride trajectories

|  | **Number of contributing individuals** | | **Assessment of model fit** | | | |
| --- | --- | --- | --- | --- | --- | --- |
|  | Total observations | Individuals with 1 measure | Mean observed, ln(trig), (SD)^a^ | Mean predicted, ln(trig), (SD)^a^ | Mean difference (observed – predicted), ln(trig)^a^ | 95% level of agreement between observed and predicted, ln(trig)^a^ |
| **Females** |  |  |  |  |  |  |
| Overall | 8458 | 3529 |  |  |  |  |
| Birth | 1474 | 1474 | -0.68 (0.45) | -0.67 (0.22) | -0.005 | -0.68 (0.45) |
| 0-9 years | 3575 | 2872 | -0.29 (0.54) | -0.30 (0.38) | 0.01 | -0.29 (0.54) |
| 9-18 years | 4883 | 2645 | -0.11 (0.41) | -0.11 (0.23) | -0.01 | -0.11 (0.41) |
| **Males** |  |  |  |  |  |  |
| Overall | 8439 | 3643 |  |  |  |  |
| Birth | 1515 | 1515 | -0.68 (0.45) | -0.68 (0.19) | -0.002 | -0.68 (0.45) |
| 0-9 years | 3730 | 3040 | -0.33 (0.53) | -0.33 (0.34) | 0.003 | -0.33 (0.53) |
| 9-18 years | 4709 | 2587 | -0.15 (0.42) | -0.15 (0.22) | -0.003 | -0.15 (0.42) |

ln(trig), natural log of triglyceride; SD, standard deviation

^a^Triglyceride is presented in the natural log and values represent the mean predicted natural log of triglyceride at each age shown.

**Table S9:** Frequencies of haplogroups among participants with at least 1 measure of each risk factor

|  | BMI  n (%) | Fat mass  n (%) | Lean mass  n (%) | SBP  n (%) | DBP  n (%) | Pulse  n (%) | HDL-c  n (%) | Non-HDL-c  n (%) | Triglycerides  n (%) |
| --- | --- | --- | --- | --- | --- | --- | --- | --- | --- |
| **Females** |  |  |  |  |  |  |  |  |  |
| **Total** | 3886 | 3216 | 3220 | 3453 | 3454 | 3455 | 3533 | 3529 | 3529 |
| **H** | 1759 (45.3) | 1449 (45.1) | 1452 (45.1) | 1570 (45.5) | 1570 (45.5) | 1571 (45.5) | 1600 (45.3) | 1598 (45.3) | 1597 (45.3) |
| **U** | 515 (13.3) | 434 (13.5) | 434 (13.5) | 457 (13.2) | 457 (13.2) | 457 (13.2) | 473 (13.4) | 472 (13.4) | 472 (13.4) |
| **T** | 401 (10.3) | 341 (10.6) | 341 (10.6) | 357 (10.3) | 357 (10.3) | 357 (10.3) | 370 (10.5) | 370 (10.5) | 368 (10.4) |
| **J** | 420 (10.8) | 353 (11.0) | 354 (11.0) | 377 (10.9) | 377 (10.9) | 377 (10.9) | 389 (11.0) | 389 (11.0) | 389 (11.0) |
| **K** | 300 (7.7) | 247 (7.7) | 247 (7.7) | 272 (7.9) | 272 (7.9) | 272 (7.9) | 277 (7.8) | 277 (7.8) | 278 (7.9) |
| **V** | 243 (6.3) | 193 (6.0) | 193 (6.0) | 209 (6.1) | 209 (6.1) | 209 (6.0) | 214 (6.1) | 214 (6.1) | 214 (6.1) |
| **W** | 67 (1.7) | 52 (1.6) | 52 (1.6) | 55 (1.6) | 55 (1.6) | 55 (1.6) | 58 (1.6) | 58 (1.6) | 58 (1.6) |
| **I** | 125 (3.2) | 99 (3.1) | 99 (3.1) | 105 (3.0) | 105 (3.0) | 105 (3.0) | 102 (2.9) | 102 (2.9) | 103 (2.9) |
| **X** | 56 (1.4) | 48 (1.5) | 48 (1.5) | 51 (1.5) | 52 (1.5) | 52 (1.5) | 50 (1.4) | 49 (1.4) | 50 (1.4) |
| **Males** |  |  |  |  |  |  |  |  |  |
| **Total** | 4068 | 3144 | 3147 | 3459 | 3459 | 3459 | 3643 | 3643 | 3643 |
| **H** | 1818 (44.7) | 1389 (44.2) | 1391 (44.2) | 1538 (44.5) | 1538 (44.5) | 1538 (44.5) | 1630 (44.7) | 1630 (44.7) | 1629 (44.7) |
| **U** | 548 (13.5) | 421 (13.4) | 421 (13.4) | 456 (13.2) | 456 (13.2) | 456 (13.2) | 484 (13.3) | 484 (13.3) | 487 (13.4) |
| **J** | 415 (10.2) | 330 (10.5) | 330 (10.5) | 362 (10.5) | 362 (10.5) | 362 (10.5) | 371 (10.2) | 371 (10.2) | 371 (10.2) |
| **T** | 448 (11.0) | 350 (11.1) | 351 (11.2) | 384 (11.1) | 384 (11.1) | 384 (11.1) | 407 (11.2) | 407 (11.2) | 407 (11.2) |
| **K** | 390 (9.6) | 299 (9.5) | 299 (9.5) | 325 (9.4) | 325 (9.4) | 325 (9.4) | 344 (9.4) | 344 (9.4) | 342 (9.4) |
| **V** | 205 (5.0) | 163 (5.2) | 163 (5.2) | 178 (5.1) | 178 (5.1) | 178 (5.1) | 185 (5.1) | 185 (5.1) | 185 (5.1) |
| **W** | 80 (2.0) | 61 (1.9) | 61 (1.9) | 71 (2.1) | 71 (2.1) | 71 (2.1) | 72 (2.0) | 72 (2.0) | 72 (2.0) |
| **I** | 101 (2.5) | 81 (2.6) | 81 (2.6) | 92 (2.7) | 92 (2.7) | 92 (2.7) | 93 (2.6) | 93 (2.6) | 93 (2.6) |
| **X** | 63 (1.5) | 50 (1.6) | 50 (1.6) | 53 (1.5) | 53 (1.5) | 53 (1.5) | 57 (1.6) | 57 (1.6) | 57 (1.6) |

**Table S10:** Number of participants with cardiometabolic risk factor measures at each time point

|  | | Birth | | Age 1 | | Age 7 | | Age 9 | | Age 10 | | Age 11 | | | Age 12 | | Age 13 | | Age 15 | | Age 18 | |
| --- | --- | --- | --- | --- | --- | --- | --- | --- | --- | --- | --- | --- | --- | --- | --- | --- | --- | --- | --- | --- | --- | --- |
| BMI ^a^ | | |  | x | x | | x | | x | | x | | x | | | x | | x | | x | |  |
| Fat mass | | |  |  |  | | 5,508 | |  | | 5,256 | |  | | | 4,605 | | 3,942 | | 3,553 | |  |
| Lean mass | | |  |  |  | | 5,518 | |  | | 5,265 | |  | | | 4,615 | | 3,955 | | 3,560 | |  |
| SBP | | |  |  | 5,915 | | 5,714 | | 5,356 | | 5,279 | | 4,996 | | |  | | 4,029 | | 3,431 | |  |
| DBP | | |  |  | 5,914 | | 5,722 | | 5,356 | | 5,284 | | 4,996 | | |  | | 4,030 | | 3,424 | |  |
| Pulse rate | | |  |  | 5,918 | | 5,719 | | 5,362 | | 5,288 | | 4,996 | | |  | | 4,031 | | 3,439 | |  |
| HDL | | | 2,989 |  | 4,387 | | 4,184 | |  | |  | |  | | |  | | 2,829 | | 2,510 | |  |
| Non-HDL | | | 2,989 |  | 4,387 | | 4,182 | |  | |  | |  | | |  | | 2,829 | | 2,510 | |  |
| Triglycerides | | | 3,015 |  | 4,366 | | 4,161 | |  | |  | |  | | |  | | 2,817 | | 2,494 | |  |
|  |  | |  | |  | |  | |  | |  | |  | | |  | |  | |  | |  |

BMI, body mass index; HDL, high density lipoprotein cholesterol; DBP, diastolic blood pressure; SBP, systolic blood pressure.

^a^ Measures available at each of these approximate ages and at several ages in between but exact timing and number of BMI measures not shown as measures were available from questionnaires, routine child health records and research clinics at different mean ages from 1 to 18 years.

**Table S11:** Characteristics at birth of the mothers of children included in models compared with those excluded due to missing exposure or outcome data

|  | **Included**  **N=6,794-7,387^a^** | **Excluded**  **N=4,673-6,037^a^** | **P-value** |
| --- | --- | --- | --- |
|  | **n (%)** | **n (%)** |  |
| **Marital Status** |  |  | <0.001 |
| Never | 1119(15.2) | 1442(23.9) |  |
| Widowed | 11(0.1) | 7(0.1) |  |
| Divorced | 286(3.9) | 284(4.7) |  |
| Separated | 100(1.4) | 116(1.9) |  |
| 1st marriage | 5336(72.5) | 3814(63.2) |  |
| 2 or 3 marriage | 505(6.9) | 374(6.2) |  |
|  |  |  |  |
| **Household Social Class** † |  |  | <0.001 |
| Professional | 1005(14.8) | 516(11.0) |  |
| Managerial/technical | 3003(44.2) | 1797(38.5) |  |
| Non manual | 1691(24.9) | 1230(26.3) |  |
| Manual | 764(11.2) | 775(16.6) |  |
| Part skilled and unskilled | 331(4.9) | 355(7.6) |  |
|  |  |  |  |
| **Maternal Education** |  |  | <0.001 |
| Less than O level | 1784(24.9) | 1932(37.2) |  |
| O level | 2502(34.9) | 1780(34.3) |  |
| A level | 1791(25.0) | 990(19.1) |  |
| Degree or above | 1099(15.3) | 492(9.5) |  |
|  |  |  |  |
| **Mother’s Partner’s Education** |  |  | <0.001 |
| Less than O level | 2075(29.8) | 2039(41.4) |  |
| O level | 1517(21.8) | 1009(20.5) |  |
| A level | 1908(27.4) | 1185(24.0) |  |
| Degree or above | 1467(21.1) | 695(14.1) |  |
|  |  |  |  |
| **Maternal Smoking during Pregnancy** |  |  | <0.001 |
| No | 5822(78.8) | 4056(69.8) |  |
| Yes | 1565(21.2) | 1755(30.2) |  |

^a^ Denominators for excluded participants in this table vary due to different rates of missing data for characteristics shown.

† Household social class was measured as the highest of the mother’s or her partner’s occupational social class using data on job title and details of occupation. Social class was derived using the standard occupational classification (SOC) codes developed by the United Kingdom Office of Population Census and Surveys and classified as I professional, II managerial and technical, IIINM non-manual, IIIM manual, and IV&V part skilled occupations and unskilled occupations.

**Table S12:** Mean trajectories of BMI and mean differences by haplogroup, estimated from multilevel models

|  | **Mean trajectory (99% CI) in haplogroup H (reference)^a^** | **Mean difference in trajectory (99% CI) comparing with haplogroup H^b^** | | | | | | | |
| --- | --- | --- | --- | --- | --- | --- | --- | --- | --- |
|  |  | **Haplogroup U** | **Haplogroup T** | **Haplogroup J** | **Haplogroup K** | **Haplogroup V** | **Haplogroup W** | **Haplogroup I** | **Haplogroup X** |
| **Females** |  |  |  |  |  |  |  |  |  |
| Age 1yr | 2.92 (2.91,2.93) | 0.02 (-2.62,2.67) | -1.24 (-4.06,1.59) | -1.83 (-4.76,1.10) | -2.07 (-5.24,1.10) | -4.02 (-7.50,-0.55) | 2.85 (-4.70,10.41) | 0.43 (-4.30,5.17) | -2.04 (-8.46,4.38) |
| Age 3yr | 2.75 (2.74,2.76) | -0.12 (-1.32,1.09) | 0.29 (-1.03,1.61) | -0.36 (-1.67,0.95) | -0.30 (-1.80,1.20) | -0.58 (-2.23,1.07) | -3.06 (-6.11,-0.02) | 0.93 (-1.32,3.19) | -2.33 (-5.46,0.81) |
| Age 7yr | 2.80 (2.79,2.81) | -0.06 (-1.60,1.47) | 0.49 (-1.21,2.19) | -0.18 (-1.84,1.48) | -0.77 (-2.66,1.13) | -2.10 (-4.18,-0.03) | -2.64 (-6.47,1.19) | 1.14 (-1.73,4.01) | -3.34 (-7.29,0.61) |
| Age 9yr | 2.85 (2.84,2.86) | -0.03 (-1.88,1.81) | 0.42 (-1.62,2.47) | -0.19 (-2.18,1.81) | -1.07 (-3.34,1.21) | -2.82 (-5.30,-0.34) | -2.03 (-6.67,2.61) | 1.25 (-2.21,4.71) | -3.55 (-8.29,1.19) |
| Age 11yr | 2.91 (2.90,2.92) | -0.01 (-2.08,2.07) | 0.31 (-1.99,2.61) | -0.19 (-2.43,2.06) | -1.30 (-3.86,1.26) | -3.22 (-6.01,-0.44) | -1.46 (-6.72,3.80) | 1.37 (-2.54,5.28) | -3.50 (-8.85,1.84) |
| Age 13yr | 2.98 (2.97,2.99) | 0.01 (-2.18,2.20) | 0.16 (-2.26,2.57) | -0.17 (-2.54,2.20) | -1.43 (-4.12,1.26) | -3.24 (-6.17,-0.31) | -1.02 (-6.59,4.56) | 1.51 (-2.62,5.65) | -3.17 (-8.84,2.49) |
| Age 15yr | 3.05 (3.04,3.06) | 0.02 (-2.20,2.25) | -0.02 (-2.47,2.42) | -0.12 (-2.54,2.29) | -1.44 (-4.18,1.30) | -2.82 (-5.82,0.17) | -0.75 (-6.46,4.95) | 1.68 (-2.54,5.91) | -2.54 (-8.36,3.28) |
| Age 18yr | 3.17 (3.16,3.19) | 0.02 (-2.52,2.55) | -0.33 (-3.07,2.41) | 0.02 (-2.76,2.80) | -1.19 (-4.31,1.93) | -1.29 (-4.78,2.20) | -0.77 (-7.39,5.86) | 2.00 (-2.86,6.86) | -0.97 (-7.77,5.82) |
| **Males** |  |  |  |  |  |  |  |  |  |
| Age 1yr | 2.91 (2.90,2.93) | 0.53 (-1.72,2.79) | -1.40 (-3.81,1.01) | 0.99 (-1.53,3.51) | -0.51 (-3.06,2.04) | 0.97 (-2.56,4.51) | -0.08 (-5.18,5.02) | 2.49 (-2.27,7.26) | 0.10 (-5.41,5.61) |
| Age 3yr | 2.77 (2.76,2.77) | -0.22 (-1.28,0.84) | 0.21 (-0.97,1.39) | -0.37 (-1.52,0.78) | 0.21 (-1.01,1.43) | -0.85 (-2.44,0.74) | 0.98 (-1.50,3.45) | 1.29 (-0.95,3.52) | 1.18 (-1.59,3.94) |
| Age 7yr | 2.79 (2.78,2.79) | 0.57 (-0.81,1.95) | -0.41 (-1.92,1.10) | -0.45 (-1.92,1.02) | 1.02 (-0.56,2.60) | -1.41 (-3.46,0.64) | 1.68 (-1.53,4.90) | 2.40 (-0.47,5.27) | 0.36 (-3.24,3.96) |
| Age 9yr | 2.83 (2.82,2.84) | 0.84 (-0.87,2.55) | -0.68 (-2.54,1.17) | -0.38 (-2.19,1.44) | 1.26 (-0.69,3.22) | -1.52 (-4.03,1.00) | 1.96 (-2.02,5.93) | 3.06 (-0.49,6.60) | 0.07 (-4.36,4.51) |
| Age 11yr | 2.88 (2.87,2.89) | 0.92 (-1.05,2.90) | -0.79 (-2.93,1.34) | -0.29 (-2.39,1.80) | 1.38 (-0.88,3.63) | -1.57 (-4.47,1.33) | 2.20 (-2.40,6.79) | 3.67 (-0.43,7.77) | -0.02 (-5.12,5.09) |
| Age 13yr | 2.95 (2.94,2.96) | 0.79 (-1.34,2.93) | -0.71 (-3.03,1.60) | -0.22 (-2.49,2.05) | 1.36 (-1.08,3.80) | -1.59 (-4.72,1.54) | 2.41 (-2.61,7.42) | 4.20 (-0.26,8.66) | 0.12 (-5.40,5.64) |
| Age 15yr | 3.02 (3.01,3.03) | 0.43 (-1.81,2.67) | -0.42 (-2.87,2.03) | -0.15 (-2.55,2.25) | 1.21 (-1.36,3.78) | -1.58 (-4.87,1.72) | 2.59 (-2.77,7.95) | 4.63 (-0.07,9.34) | 0.52 (-5.29,6.33) |
| Age 18yr | 3.16 (3.15,3.17) | -0.58 (-3.23,2.06) | 0.48 (-2.47,3.42) | -0.09 (-2.97,2.78) | 0.71 (-2.33,3.74) | -1.51 (-5.41,2.40) | 2.81 (-3.72,9.34) | 5.06 (-0.47,10.60) | 1.63 (-5.22,8.48) |

BMI, body mass index; CI, confidence interval; yr, years

^a^ BMI is presented in the natural log and values represent the mean predicted natural log of BMI at each age shown.

^b^ Differences at each age are back transformed from the log scale and are interpreted as the percentage difference in the mean level in original units at each age comparing each category with the reference trajectory.

**Table S13:** Mean trajectories of fat mass and mean differences by haplogroup, estimated from multilevel models

|  | **Mean log fat mass trajectory (99% CI) in haplogroup H (kg or kg/yr** **) (reference)^a^** | **Mean difference in fat mass trajectory (99% CI) comparing with haplogroup H(% or %/yr)^b^** | | | | | | | |
| --- | --- | --- | --- | --- | --- | --- | --- | --- | --- |
|  |  | **Haplogroup U** | **Haplogroup T** | **Haplogroup J** | **Haplogroup K** | **Haplogroup V** | **Haplogroup W** | **Haplogroup I** | **Haplogroup X** |
| **Female** |  |  |  |  |  |  |  |  |  |
| Age 9yr (kg) or (%) | 2.02 (1.98,2.05) | -0.82 (-8.37,6.73) | 1.12 (-7.33,9.58) | 1.09 (-7.24,9.42) | -1.77 (-11.12,7.58) | -9.33 (-19.03,0.38) | -12.37 (-29.64,4.89) | 2.70 (-12.14,17.53) | -16.39 (-33.30,0.53) |
| Change 9-13yr (kg/yr) or (%/yr) | 0.15 (0.15,0.16) | -0.06 (-1.48,1.37) | -0.54 (-2.09,1.02) | 0.08 (-1.48,1.64) | -0.76 (-2.52,1.01) | -0.23 (-2.23,1.77) | 0.99 (-2.77,4.75) | -0.81 (-3.48,1.85) | 0.94 (-2.85,4.73) |
| Change 13-15yr (kg/yr) or (%/yr) | 0.10 (0.09,0.11) | 0.56 (-2.04,3.15) | -0.78 (-3.60,2.04) | -0.21 (-3.01,2.59) | 0.82 (-2.46,4.09) | 1.70 (-2.05,5.45) | 3.10 (-3.72,9.91) | 0.78 (-4.11,5.66) | 3.81 (-3.55,11.17) |
| Change 15-18yr (kg/yr) or (%/yr) | 0.06 (0.05,0.07) | -0.99 (-2.80,0.81) | -0.32 (-2.23,1.60) | 0.11 (-1.84,2.05) | 0.29 (-1.92,2.50) | 1.86 (-0.79,4.50) | -0.46 (-5.20,4.28) | -0.13 (-3.58,3.32) | 1.01 (-4.01,6.03) |
| Age 18yr (kg) or (%) | 3.01 (2.98,3.04) | -2.88 (-9.26,3.49) | -3.49 (-10.29,3.30) | 1.21 (-9.36,11.77) | -4.40 (-15.85,7.05) | 0.40 (-13.31,14.11) | 6.70 (-20.17,33.57) | -1.95 (-19.89,15.99) | 8.90 (-19.56,37.36) |
| **Male** |  |  |  |  |  |  |  |  |  |
| Age 9yr (kg) or (%) | 1.70 (1.65,1.74) | 1.67 (-7.73,11.08) | -4.63 (-14.22,4.96) | 2.70 (-7.52,12.93) | -0.41 (-10.92,10.09) | -2.81 (-16.29,10.68) | 1.49 (-20.25,23.23) | 10.76 (-10.06,31.57) | -10.46 (-31.84,10.92) |
| Change 9-13yr (kg/yr) or (%/yr) | 0.15 (0.14,0.16) | -0.02 (-2.05,2.01) | 0.08 (-2.13,2.30) | -1.01 (-3.21,1.19) | 0.65 (-1.67,2.96) | 0.76 (-2.38,3.90) | 1.05 (-3.78,5.88) | 1.61 (-2.44,5.66) | 3.57 (-1.70,8.84) |
| Change 13-15yr (kg/yr) or (%/yr) | -0.08 (-0.09,-0.06) | -0.65 (-4.37,3.07) | 0.85 (-3.29,5.00) | -0.62 (-4.66,3.42) | -2.92 (-6.97,1.12) | -3.73 (-9.39,1.93) | 4.18 (-5.14,13.49) | 3.44 (-3.88,10.76) | 2.48 (-6.44,11.40) |
| Change 15-18yr (kg/yr) or (%/yr) | 0.10 (0.08,0.11) | -0.40 (-3.43,2.62) | 2.48 (-0.91,5.88) | 0.76 (-2.53,4.04) | 1.60 (-1.84,5.03) | 1.43 (-3.27,6.13) | -4.18 (-10.96,2.59) | -3.45 (-9.27,2.37) | 0.55 (-6.81,7.91) |
| Age 18yr (kg) or (%) | 2.46 (2.40,2.51) | -0.92 (-11.85,10.02) | 4.75 (-8.05,17.56) | -7.16 (-23.13,8.81) | -1.72 (-19.55,16.11) | -6.71 (-28.99,15.57) | 15.50 (-28.72,59.71) | 31.80 (-9.63,73.23) | 37.72 (-17.44,92.87) |

CI, confidence interval; kg/yr, kilograms per year; %/yr, percentage per year.

^a^Fat mass was transformed using the natural log. All predicted mean values (kg) and rates of change per year (kg/yr) are on the log scale

^b^The difference between haplogroups is back transformed from the log scale for ease of interpretation and is interpreted as the percentage difference in the mean level comparing each category with haplogroup H or percentage difference in change per year (%/yr) comparing each category with haplogroup H.

**Table S14:** Mean trajectories of lean mass and mean differences by haplogroup, estimated from multilevel models

|  | **Mean trajectory (99% CI) in haplogroup H (reference)** | **Mean difference in trajectory (99% CI) comparing with haplogroup H** | | | | | | | |
| --- | --- | --- | --- | --- | --- | --- | --- | --- | --- |
|  |  | **Haplogroup U** | **Haplogroup T** | **Haplogroup J** | **Haplogroup K** | **Haplogroup V** | **Haplogroup W** | **Haplogroup I** | **Haplogroup X** |
| **Female** |  |  |  |  |  |  |  |  |  |
| Age 9yr (kg) | 20.92 (20.71,21.12) | -0.25 (-0.68,0.18) | -0.21 (-0.68,0.26) | -0.004 (-0.47,0.46) | -0.37 (-0.90,0.16) | -0.39 (-0.99,0.21) | -0.99 (-2.11,0.12) | 0.14 (-0.67,0.96) | -1.34 (-2.48,-0.20) |
| Change 9-13yr (kg/yr) | 3.17 (3.10,3.23) | 0.02 (-0.12,0.15) | 0.10 (-0.05,0.25) | 0.09 (-0.06,0.23) | 0.01 (-0.157,0.18) | -0.09 (-0.29,0.10) | 0.18 (-0.17,0.53) | -0.07 (-0.33,0.18) | -0.03 (-0.39,0.34) |
| Change 13-15yr (kg/yr) | 1.72 (1.57,1.87) | 0.18 (-0.12,0.48) | -0.09 (-0.42,0.24) | -0.11 (-0.44,0.22) | 0.07 (-0.31,0.44) | 0.17 (-0.26,0.61) | -0.04 (-0.81,0.73) | 0.44 (-0.13,1.01) | -0.01 (-0.84,0.82) |
| Change 15-18yr (kg/yr) | 0.40 (0.33,0.47) | -0.07 (-0.22,0.08) | -0.04 (-0.19,0.11) | -0.04 (-0.19,0.12) | 0.01 (-0.17,0.19) | -0.18 (-0.39,0.03) | 0.13 (-0.25,0.51) | -0.19 (-0.47,0.09) | 0.02 (-0.38,0.43) |
| Age 18yr (kg) | 38.25 (37.93,38.57) | -0.01 (-0.68,0.65) | -0.10 (-0.82,0.61) | 0.01 (-0.71,0.73) | -0.16 (-0.98,0.66) | -0.97 (-1.91,-0.02) | 0.04 (-1.71,1.79) | 0.15 (-1.11,1.41) | -1.41 (-3.24,0.42) |
|  |  |  |  |  |  |  |  |  |  |
| **Male** |  |  |  |  |  |  |  |  |  |
| Age 9yr (kg) | 22.88 (22.67,23.09) | 0.06 (-0.38,0.50) | 0.02 (-0.46,0.50) | -0.13 (-0.61,0.35) | 0.20 (-0.30,0.71) | -0.45 (-1.13,0.23) | -0.05 (-1.08,0.98) | 0.11 (-0.79,1.00) | 0.53 (-0.60,1.65) |
| Change 9-13yr (kg/yr) | 2.79 (2.67,2.90) | 0.09 (-0.15,0.32) | 0.17 (-0.09,0.43) | 0.08 (-0.18,0.34) | 0.09 (-0.18,0.36) | -0.01 (-0.37,0.35) | 0.09 (-0.48,0.65) | 0.26 (-0.20,0.73) | -0.19 (-0.79,0.41) |
| Change 13-15yr (kg/yr) | 7.34 (7.15,7.53) | -0.15 (-0.55,0.24) | -0.03 (-0.47,0.40) | -0.005 (-0.44,0.43) | 0.23 (-0.21,0.67) | 0.15 (-0.48,0.78) | -0.62 (-1.57,0.34) | -0.13 (-0.88,0.62) | 0.13 (-0.79,1.05) |
| Change 15-18yr (kg/yr) | 2.25 (2.08,2.42) | 0.01 (-0.34,0.37) | -0.14 (-0.53,0.25) | -0.13 (-0.52,0.25) | -0.17 (-0.56,0.23) | 0.0003 (-0.54,0.54) | 0.15 (-0.68,0.99) | -0.05 (-0.76,0.66) | -0.45 (-1.33,0.42) |
| Age 18yr (kg) | 55.45 (54.93,55.97) | 0.15 (-0.94,1.23) | 0.21 (-0.99,1.41) | -0.22 (-1.39,0.96) | 0.52 (-0.72,1.75) | -0.19 (-1.81,1.43) | -0.47 (-3.03,2.09) | 0.74 (-1.45,2.93) | -1.32 (-4.04,1.40) |

kg/yr, kilograms per year; yr

**Table S15:** Mean trajectories of SBP, DBP and pulse rate and mean differences by haplogroup, estimated from multilevel models

|  | **Mean trajectory (99% CI) in haplogroup H (reference)^a^** | **Mean difference in trajectory (99% CI) comparing with haplogroup H^a^** | | | | | | | |
| --- | --- | --- | --- | --- | --- | --- | --- | --- | --- |
|  |  | **Haplogroup U** | **Haplogroup T** | **haplogroup J** | **haplogroup K** | **Haplogroup V** | **Haplogroup W** | **Haplogroup I** | **Haplogroup X** |
| **SBP**  **Females** |  |  |  |  |  |  |  |  |  |
| Age 7yr (mmHg) | 98.32 (97.65,98.99) | -0.37 (-1.77,1.02) | -0.73 (-2.26,0.81) | -0.57 (-2.09,0.96) | -0.47 (-2.20,1.27) | -1.18 (-3.16,0.80) | 0.27 (-3.46,3.99) | -0.36 (-3.02,2.29) | -3.36 (-7.07,0.36) |
| Change 7-12yr (mmHg/yr) | 1.82 (1.66,1.98) | 0.07 (-0.26,0.39) | 0.20 (-0.16,0.56) | 0.10 (-0.27,0.46) | -0.12 (-0.54,0.29) | 0.02 (-0.45,0.48) | -0.39 (-1.25,0.47) | -0.12 (-0.75,0.51) | 0.64 (-0.23,1.52) |
| Change 12-16yr (mmHg/yr) | 3.89 (3.62,4.15) | -0.23 (-0.77,0.32) | -0.36 (-0.94,0.23) | -0.07 (-0.66,0.51) | 0.27 (-0.41,0.95) | 0.24 (-0.52,1.00) | 0.66 (-0.77,2.09) | 0.27 (-0.76,1.29) | -0.32 (-1.81,1.17) |
| Change 16-18yr (mmHg/yr) | -6.04 (-6.62,-5.47) | 0.53 (-0.66,1.73) | 0.64 (-0.60,1.89) | 0.30 (-0.99,1.59) | 0.08 (-1.42,1.58) | -0.02 (-1.70,1.67) | 0.85 (-2.25,3.95) | 0.16 (-2.06,2.38) | -0.24 (-3.46,2.97) |
| Age 18yr (mmHg) | 110.91 (110.14,111.67) | 0.12 (-1.46,1.70) | 0.15 (-1.52,1.81) | 0.22 (-1.52,1.97) | 0.14 (-1.83,2.10) | -0.17 (-2.49,2.15) | 2.68 (-1.49,6.85) | 0.40 (-2.66,3.46) | -1.89 (-6.26,2.47) |
| **Males** |  |  |  |  |  |  |  |  |  |
| Age 7yr (mmHg) | 97.96 (97.31,98.60) | 0.07 (-1.29,1.43) | -0.63 (-2.10,0.84) | -0.37 (-1.83,1.08) | 0.19 (-1.35,1.73) | 0.45 (-1.60,2.49) | -0.74 (-3.74,2.26) | -0.24 (-2.95,2.48) | -1.96 (-5.47,1.54) |
| Change 7-12yr (mmHg/yr) | 1.58 (1.42,1.74) | 0.12 (-0.21,0.44) | 0.18 (-0.17,0.53) | 0.12 (-0.23,0.47) | -0.05 (-0.41,0.32) | -0.19 (-0.67,0.30) | -0.06 (-0.79,0.67) | 0.83 (0.18,1.47) | 0.58 (-0.25,1.41) |
| Change 12-16yr (mmHg/yr) | 5.82 (5.55,6.08) | -0.16 (-0.70,0.39) | -0.16 (-0.75,0.44) | 0.23 (-0.36,0.82) | 0.23 (-0.37,0.82) | 0.37 (-0.46,1.20) | -0.10 (-1.42,1.22) | -1.23 (-2.27,-0.19) | -0.47 (-1.77,0.82) |
| Change 16-18yr (mmHg/yr) | -3.72 (-4.35,-3.09) | -0.07 (-1.39,1.24) | 0.20 (-1.23,1.62) | -0.58 (-1.98,0.83) | 0.23 (-1.18,1.65) | -1.22 (-3.16,0.72) | -1.05 (-4.10,1.99) | 0.45 (-2.21,3.11) | 0.37 (-2.73,3.48) |
| Age 18yr (mmHg) | 121.68 (120.74,122.63) | -0.10 (-2.06,1.87) | 0.01 (-2.12,2.15) | 0.02 (-2.08,2.12) | 1.34 (-0.83,3.51) | -1.44 (-4.28,1.39) | -3.54 (-8.00,0.91) | -0.12 (-4.16,3.92) | -0.23 (-5.07,4.62) |
| **DBP**  **Females** |  |  |  |  |  |  |  |  |  |
| Age 7yr (mmHg) | 57.17 (56.70,57.64) | -0.50 (-1.48,0.48) | -0.32 (-1.40,0.76) | -0.23 (-1.30,0.85) | -0.67 (-1.89,0.54) | -0.25 (-1.65,1.14) | -1.74 (-4.37,0.89) | 0.41 (-1.45,2.28) | -1.49 (-4.10,1.12) |
| Change 7-12yr (mmHg/yr) | 0.07 (-0.05,0.19) | 0.16 (-0.09,0.41) | 0.09 (-0.18,0.36) | -0.01 (-0.28,0.26) | 0.04 (-0.27,0.35) | -0.01 (-0.37,0.34) | 0.34 (-0.32,0.99) | -0.15 (-0.63,0.32) | 0.24 (-0.42,0.89) |
| Change 12-16yr (mmHg/yr) | 2.45 (2.24,2.67) | -0.20 (-0.65,0.25) | -0.26 (-0.74,0.22) | 0.19 (-0.29,0.66) | -0.20 (-0.76,0.36) | -0.18 (-0.80,0.44) | -0.58 (-1.75,0.60) | -0.16 (-1.00,0.68) | -0.14 (-1.36,1.08) |
| Change 16-18yr (mmHg/yr) | -1.30 (-1.77,-0.83) | 0.27 (-0.70,1.25) | 0.45 (-0.57,1.47) | -0.60 (-1.66,0.47) | 0.31 (-0.93,1.55) | 0.76 (-0.61,2.12) | 1.88 (-0.69,4.46) | 0.52 (-1.26,2.31) | 0.52 (-2.11,3.15) |
| Age 18yr (mmHg) | 64.72 (64.16,65.28) | 0.05 (-1.10,1.20) | -0.01 (-1.22,1.19) | -0.74 (-2.02,0.55) | -0.65 (-2.10,0.79) | 0.47 (-1.21,2.16) | 1.40 (-1.66,4.45) | 0.06 (-2.14,2.26) | 0.18 (-3.01,3.37) |
| **Males** |  |  |  |  |  |  |  |  |  |
| Age 7yr (mmHg) | 56.12 (55.64,56.60) | 0.28 (-0.73,1.29) | 0.03 (-1.06,1.12) | 0.22 (-0.86,1.30) | -0.13 (-1.27,1.01) | -0.08 (-1.60,1.44) | 0.12 (-2.10,2.34) | 0.54 (-1.47,2.56) | -0.66 (-3.25,1.94) |
| Change 7-12yr (mmHg/yr) | 0.14 (0.02,0.26) | 0.05 (-0.21,0.31) | 0.02 (-0.26,0.30) | 0.02 (-0.25,0.30) | -0.07 (-0.35,0.22) | 0.17 (-0.21,0.56) | -0.01 (-0.59,0.56) | 0.20 (-0.31,0.71) | 0.05 (-0.61,0.71) |
| Change 12-16yr (mmHg/yr) | 2.88 (2.63,3.13) | 0.13 (-0.39,0.65) | 0.00 (-0.57,0.56) | -0.10 (-0.66,0.47) | 0.05 (-0.52,0.62) | -0.28 (-1.07,0.50) | -0.46 (-1.71,0.80) | -0.30 (-1.30,0.70) | 0.37 (-0.86,1.61) |
| Change 16-18yr (mmHg/yr) | -2.65 (-3.22,-2.08) | -0.62 (-1.81,0.57) | 0.07 (-1.23,1.37) | 0.10 (-1.19,1.38) | 0.58 (-0.70,1.87) | 0.01 (-1.75,1.77) | 1.08 (-1.72,3.88) | -0.36 (-2.82,2.11) | -0.19 (-3.02,2.64) |
| Age 18yr (mmHg) | 63.03 (62.35,63.71) | -0.22 (-1.62,1.18) | 0.26 (-1.27,1.79) | 0.15 (-1.36,1.65) | 0.91 (-0.62,2.44) | -0.34 (-2.34,1.66) | 0.39 (-2.76,3.54) | -0.35 (-3.39,2.68) | 0.70 (-2.76,4.16) |
| **Pulse rate**  **Females** |  |  |  |  |  |  |  |  |  |
| Age 7yr (bpm) | 85.72 (84.95,86.48) | -0.33 (-1.93,1.26) | 0.31 (-1.45,2.06) | 0.79 (-0.96,2.54) | 0.92 (-1.06,2.89) | -0.50 (-2.76,1.76) | 2.32 (-1.94,6.58) | 0.64 (-2.38,3.67) | -0.43 (-4.65,3.80) |
| Change 7-12yr (bpm/yr) | -1.79 (-1.97,-1.61) | 0.18 (-0.20,0.55) | -0.07 (-0.48,0.34) | -0.11 (-0.52,0.31) | 0.04 (-0.43,0.51) | 0.11 (-0.43,0.64) | -0.55 (-1.54,0.44) | -0.18 (-0.90,0.54) | -0.09 (-1.09,0.91) |
| Change 12-16yr (bpm/yr) | -0.27 (-0.55,0.02) | 0.020 (-0.56,0.60) | -0.15 (-0.77,0.47) | 0.01 (-0.61,0.63) | -0.40 (-1.13,0.32) | 0.02 (-0.78,0.83) | 0.25 (-1.26,1.76) | -0.06 (-1.15,1.03) | 0.86 (-0.72,2.45) |
| Change 16-18yr (bpm/yr) | -4.76 (-5.37,-4.14) | 0.49 (-0.78,1.77) | 0.30 (-1.03,1.63) | -0.75 (-2.13,0.64) | 0.43 (-1.18,2.04) | 0.58 (-1.23,2.38) | 0.05 (-3.27,3.36) | -0.22 (-2.59,2.15) | -1.97 (-5.43,1.49) |
| Age 18yr (bpm) | 66.19 (65.30,67.08) | 1.63 (-0.21,3.47) | -0.03 (-1.96,1.90) | -1.18 (-3.22,0.85) | 0.35 (-1.94,2.64) | 1.28 (-1.41,3.97) | 0.64 (-4.24,5.52) | -0.95 (-4.49,2.59) | -1.35 (-6.43,3.73) |
| **Males** |  |  |  |  |  |  |  |  |  |
| Age 7yr (bpm) | 83.02 (82.27,83.77) | -0.05 (-1.63,1.54) | -0.92 (-2.63,0.80) | 0.54 (-1.16,2.24) | -0.16 (-1.95,1.63) | 1.09 (-1.29,3.48) | 0.51 (-2.98,4.00) | 0.50 (-2.67,3.67) | -3.16 (-7.25,0.93) |
| Change 7-12yr (bpm/yr) | -1.91 (-2.09,-1.72) | -0.04 (-0.44,0.35) | 0.30 (-0.12,0.72) | 0.07 (-0.35,0.49) | 0.00 (-0.44,0.44) | 0.12 (-0.46,0.70) | -0.42 (-1.30,0.46) | 0.17 (-0.61,0.95) | 0.67 (-0.34,1.67) |
| Change 12-16yr (bpm/yr) | -0.75 (-1.04,-0.47) | -0.02 (-0.62,0.58) | -0.38 (-1.03,0.28) | 0.06 (-0.59,0.70) | -0.22 (-0.87,0.43) | -0.30 (-1.21,0.62) | 0.56 (-0.88,2.01) | 0.14 (-1.00,1.28) | -0.11 (-1.54,1.33) |
| Change 16-18yr (bpm/yr) | -4.16 (-4.82,-3.50) | -0.12 (-1.51,1.26) | 0.06 (-1.45,1.57) | -0.34 (-1.83,1.14) | 0.29 (-1.19,1.77) | -0.41 (-2.44,1.62) | -0.48 (-3.69,2.73) | -1.20 (-4.06,1.66) | 0.03 (-3.27,3.33) |
| Age 18yr (bpm) | 62.15 (61.18,63.11) | -0.58 (-2.58,1.42) | -0.81 (-2.99,1.37) | 0.42 (-1.72,2.56) | -0.44 (-2.64,1.75) | -0.31 (-3.16,2.55) | -0.31 (-4.84,4.21) | -0.48 (-4.67,3.71) | -0.19 (-5.12,4.75) |

DBP, diastolic blood pressure; SBP, systolic blood pressure; CI, confidence interval

^a^Units are presented in mmHg for SBP and DBP and bpm for pulse rate

**Table S16:** Mean trajectories of HDL-c and non-HDL-c and mean differences by haplogroup, estimated from multilevel models

|  | **Mean trajectory (99% CI) in haplogroup H (reference)** | **Mean difference in trajectory (99% CI) comparing with haplogroup H** | | | | | | | |
| --- | --- | --- | --- | --- | --- | --- | --- | --- | --- |
|  |  | **Haplogroup U** | **Haplogroup T** | **Haplogroup J** | **Haplogroup K** | **Haplogroup V** | **Haplogroup W** | **Haplogroup I** | **Haplogroup X** |
| **HDL-c**  **Females** |  |  |  |  |  |  |  |  |  |
| Birth (mmol/l) | 0.79 (0.39,1.19) | -0.24 (-1.07,0.58) | 0.12 (-0.78,1.02) | 0.12 (-0.75,0.99) | 0.13 (-0.95,1.21) | 0.37 (-0.75,1.49) | -0.23 (-2.32,1.85) | 1.23 (-0.52,2.98) | -0.25 (-2.63,2.13) |
| Change 0-7yr (mmol/l/yr) | 0.09 (0.04,0.15) | 0.04 (-0.08,0.16) | -0.02 (-0.15,0.11) | -0.02 (-0.14,0.11) | -0.01 (-0.17,0.14) | -0.05 (-0.21,0.11) | 0.04 (-0.26,0.34) | -0.18 (-0.43,0.07) | 0.03 (-0.31,0.37) |
| Change 7-18yr (mmol/l/yr) | -0.01 (-0.01,-0.01) | 0.0003 (-0.01,0.01) | 0.0003 (-0.01,0.01) | -0.005 (-0.01,0.001) | -0.006 (-0.01,0.0001) | 0.0002 (-0.01,0.01) | -0.01 (-0.02,0.01) | -0.004 (-0.02,0.01) | 0.005 (-0.01,0.02) |
| Age 18yr (mmol/l) | 1.33 (1.30,1.35) | 0.02 (-0.03,0.08) | -0.003 (-0.06,0.05) | -0.04 (-0.10,0.02) | -0.04 (-0.11,0.02) | 0.05 (-0.02,0.13) | -0.07 (-0.21,0.08) | -0.05 (-0.16,0.05) | 0.02 (-0.13,0.16) |
| **Males** |  |  |  |  |  |  |  |  |  |
| Birth (mmol/l) | 0.81 (0.52,1.10) | -0.25 (-0.86,0.36) | -0.33 (-1.01,0.35) | -0.32 (-0.98,0.35) | -0.31 (-1.02,0.40) | -0.34 (-1.21,0.52) | 2.13 (0.75,3.51) | -0.30 (-1.65,1.06) | -0.38 (-2.03,1.27) |
| Change 0-7yr (mmol/l/yr) | 0.10 (0.06,0.15) | 0.03 (-0.05,0.12) | 0.05 (-0.05,0.15) | 0.04 (-0.05,0.14) | 0.05 (-0.05,0.15) | 0.05 (-0.08,0.17) | -0.30 (-0.50,-0.11) | 0.05 (-0.14,0.25) | 0.05 (-0.19,0.28) |
| Change 7-18yr (mmol/l/yr) | -0.04 (-0.04,-0.03) | 0.003 (-0.002,0.01) | 0.001 (-0.004,0.007) | 0.003 (-0.002,0.01) | -0.0004 (-0.01,0.01) | 0.001 (-0.01,0.01) | 0.004 (-0.01,0.02) | -0.004 (-0.01,0.01) | 0.003 (-0.01,0.01) |
| Age 18yr (mmol/l) | 1.14 (1.11,1.16) | 0.02 (-0.03,0.06) | 0.04 (-0.02,0.09) | 0.01 (-0.04,0.06) | 0.03 (-0.02,0.08) | -0.01 (-0.08,0.06) | 0.05 (-0.06,0.17) | 0.01 (-0.08,0.10) | -0.01 (-0.12,0.11) |
| **Non-HDL-c**  **Females** |  |  |  |  |  |  |  |  |  |
| Birth (mmol/l) | 1.68 (1.37,1.98) | -0.04 (-0.68,0.61) | -0.12 (-0.82,0.58) | -0.16 (-0.85,0.52) | 0.24 (-0.57,1.05) | -0.42 (-1.31,0.47) | 0.58 (-1.06,2.22) | -0.47 (-1.76,0.82) | 0.29 (-1.54,2.11) |
| Change 0-9yr (mmol/l/yr) | 0.16 (0.13,0.20) | 0.003 (-0.08,0.08) | 0.01 (-0.08,0.10) | 0.009 (-0.08,0.09) | -0.03 (-0.13,0.07) | 0.04 (-0.07,0.15) | -0.06 (-0.27,0.14) | 0.05 (-0.11,0.21) | -0.04 (-0.27,0.19) |
| Change 9-18yr (mmol/l/yr) | -0.07 (-0.08,-0.06) | 0.005 (-0.01,0.02) | -0.004 (-0.02,0.01) | 0.003 (-0.02,0.02) | 0.01 (-0.01,0.03) | 0.01 (-0.02,0.03) | 0.01 (-0.04,0.06) | -0.01 (-0.05,0.02) | 0.04 (-0.01,0.09) |
| Age 18yr (mmol/l) | 2.50 (2.44,2.55) | 0.03 (-0.09,0.15) | -0.05 (-0.18,0.08) | -0.06 (-0.19,0.07) | 0.04 (-0.11,0.18) | -0.001 (-0.17,0.17) | 0.11 (-0.24,0.47) | -0.13 (-0.37,0.11) | 0.28 (-0.05,0.61) |
| **Males** |  |  |  |  |  |  |  |  |  |
| Birth (mmol/l) | 1.40 (1.16,1.64) | 0.23 (-0.27,0.72) | 0.29 (-0.26,0.83) | 0.22 (-0.32,0.75) | 0.29 (-0.28,0.86) | 0.14 (-0.58,0.86) | -1.54 (-2.68,-0.40) | 0.30 (-0.78,1.37) | 0.11 (-1.17,1.39) |
| Change 0-9yr (mmol/l/yr) | 0.17 (0.14,0.20) | -0.03 (-0.09,0.03) | -0.03 (-0.10,0.03) | -0.03 (-0.09,0.04) | -0.03 (-0.10,0.04) | -0.01 (-0.10,0.08) | 0.19 (0.05,0.33) | -0.035 (-0.16,0.09) | -0.005 (-0.16,0.15) |
| Change 9-18yr (mmol/l/yr) | -0.07 (-0.08,-0.06) | 0.01 (-0.01,0.02) | 0.01 (-0.004,0.03) | 0.01 (-0.01,0.02) | 0.01 (-0.01,0.02) | -0.002 (-0.02,0.02) | -0.003 (-0.04,0.03) | 0.002 (-0.03,0.03) | -0.001 (-0.03,0.03) |
| Age 18yr (mmol/l) | 2.27 (2.21,2.32) | 0.03 (-0.09,0.14) | 0.07 (-0.05,0.20) | 0.05 (-0.07,0.17) | 0.10 (-0.02,0.23) | 0.02 (-0.15,0.20) | 0.16 (-0.12,0.45) | -0.002 (-0.22,0.22) | 0.06 (-0.21,0.34) |

CI, confidence interval; HDL-c, high density lipoprotein cholesterol; mmol/l, millimole per litre; mmol/l, millimole per litre per year

**Table S17:** Mean trajectories of triglyceride estimated from multilevel models**,** by haplogroup

|  | **Mean trajectory (99% CI) in haplogroup H (reference)^a^** | **Mean difference in trajectory (99% CI) comparing with haplogroup H^b^** | | | | | | | |
| --- | --- | --- | --- | --- | --- | --- | --- | --- | --- |
|  |  | **Haplogroup U** | **Haplogroup T** | **Haplogroup J** | **Haplogroup K** | **Haplogroup V** | **Haplogroup W** | **Haplogroup I** | **Haplogroup X** |
| **Female** |  |  |  |  |  |  |  |  |  |
| Birth (mmol/l or %) | -0.67 (-0.71,-0.63) | 1.20 (-8.01,10.41) | -1.52 (-11.28,8.24) | 0.88 (-8.82,10.58) | -0.58 (-12.33,11.17) | -5.67 (-17.37,6.02) | 10.05 (-15.40,35.50) | -2.67 (-21.22,15.88) | 9.84 (-19.05,38.73) |
| Change 0-9yr (mmol/l/yr or %/yr) | 0.09 (0.08,0.09) | 0.03 (-1.27,1.34) | 0.52 (-0.92,1.95) | -0.25 (-1.62,1.13) | -0.43 (-2.09,1.22) | 0.45 (-1.36,2.26) | -1.70 (-4.94,1.54) | 0.59 (-2.11,3.28) | -1.32 (-4.94,2.29) |
| Change 9-18yr (mmol/l/yr or %/yr) | -0.05 (-0.05,-0.04) | 0.07 (-0.93,1.06) | -0.17 (-1.21,0.88) | 0.63 (-0.44,1.69) | 0.88 (-0.31,2.06) | 0.74 (-0.70,2.17) | 1.29 (-1.53,4.10) | -0.03 (-1.97,1.91) | 1.06 (-1.65,3.77) |
| Age 18yr (mmol/l or %) | -0.31 (-0.35,-0.28) | 2.09 (-4.83,9.02) | 1.61 (-5.53,8.74) | 4.36 (-3.22,11.94) | 3.42 (-4.83,11.66) | 4.94 (-5.09,14.97) | 5.84 (-14.92,26.60) | 2.30 (-11.21,15.81) | 7.13 (-12.82,27.08) |
| **Male** |  |  |  |  |  |  |  |  |  |
| Birth (mmol/l or %) | -0.70 (-0.74,-0.65) | 2.58 (-6.67,11.83) | 1.96 (-8.36,12.28) | 5.04 (-5.28,15.35) | 1.89 (-8.84,12.63) | 6.39 (-7.26,20.04) | 7.48 (-14.52,29.48) | 9.11 (-12.82,31.03) | -1.56 (-26.11,23.00) |
| Change 0-9yr (mmol/l/yr or %/yr) | 0.08 (0.07,0.09) | -0.34 (-1.64,0.95) | 0.03 (-1.41,1.47) | -0.81 (-2.19,0.58) | -0.25 (-1.75,1.25) | -0.15 (-2.01,1.71) | -1.17 (-4.09,1.75) | -0.87 (-3.63,1.90) | 0.22 (-3.28,3.72) |
| Change 9-18yr (mmol/l/yr or %/yr) | -0.04 (-0.04,-0.03) | -0.28 (-1.37,0.81) | -0.18 (-1.36,1.00) | 0.20 (-0.96,1.35) | 0.44 (-0.77,1.66) | 0.20 (-1.49,1.88) | 1.50 (-1.17,4.17) | -0.32 (-2.41,1.78) | 0.61 (-2.09,3.31) |
| Age 18yr (mmol/l or %) | -0.33 (-0.36,-0.29) | -3.02 (-10.31,4.27) | 0.62 (-7.65,8.89) | -0.61 (-8.57,7.34) | 3.68 (-4.97,12.33) | 6.83 (-5.69,19.36) | 10.55 (-10.62,31.71) | -1.93 (-15.91,12.06) | 6.06 (-13.18,25.31) |

CI, confidence interval; mmol/l, millimole per litre; mmol/l/year, millimoles per litre per year; %/yr, percentage per year

^a^Triglyceride was transformed using the natural log. All predicted mean values (mmol/l) and rates of change per year (mmol/l/yr) are on the log scale

^b^The difference between haplogroups is back transformed from the log scale for ease of interpretation and is interpreted as the percentage difference in the mean level comparing each category with haplogroup H or percentage difference in change per year (%/yr) comparing each category with haplogroup H

**References:**

1. Purcell S, Neale B, Todd-Brown K, Thomas L, Ferreira MAR, Bender D, et al. PLINK: A tool set for whole-genome association and population-based linkage analyses. Am J Hum Genet. 2007;81(3):559–75.

2. Kloss-Brandstätter A, Pacher D, Schönherr S, Weissensteiner H, Binna R, Specht G, et al. HaploGrep: A fast and reliable algorithm for automatic classification of mitochondrial DNA haplogroups. Hum Mutat. 2011;32(1):25–32.

3. Staley JR, Bradley J, Silverwood RJ, Howe LD, Tilling K, Lawlor DA, et al. Associations of blood pressure in pregnancy with offspring blood pressure trajectories during childhood and adolescence: findings from a prospective study. J Am Heart Assoc. 2015;4(5):1–12.

4. Morris TT, Northstone K, Howe LD. Examining the association between early life social adversity and BMI changes in childhood: a life course trajectory analysis. Pediatr Obes. 2016;11(4):306–12.

5. O’Keeffe LM, Simpkin AJ, Tilling K, Anderson EL, Hughes AD, Lawlor DA, et al. Sex-specific trajectories of measures of cardiovascular health during childhood and adolescence: A prospective cohort study. Atherosclerosis. 2018;278(September):190–6.

6. O’Keeffe LM, Simpkin AJ, Tilling K, Anderson EL, Hughes AD, Lawlor DA, et al. Data on trajectories of measures of cardiovascular health in the Avon Longitudinal Study of Parents and Children (ALSPAC). Data Br. 2019;23:103687.
